# ATRX guards against aberrant differentiation in mesenchymal progenitor cells

**DOI:** 10.1093/nar/gkae160

**Published:** 2024-03-13

**Authors:** Yan Fang, Douglas Barrows, Yakshi Dabas, Thomas S Carroll, Sam Singer, William D Tap, Benjamin A Nacev

**Affiliations:** Department of Medicine, Memorial Sloan Kettering Cancer Center, New York, NY10065, USA; Laboratory of Chromatin Biology and Epigenetics, The Rockefeller University, New York, NY 10065, USA; Bioinformatics Resource Center, The Rockefeller University, New York, NY10065, USA; Laboratory of Chromatin Biology and Epigenetics, The Rockefeller University, New York, NY 10065, USA; Bioinformatics Resource Center, The Rockefeller University, New York, NY10065, USA; Department of Surgery, Memorial Sloan Kettering Cancer Center, New York, NY10065, USA; Department of Medicine, Memorial Sloan Kettering Cancer Center, New York, NY10065, USA; Department of Medicine, University of Pittsburgh, Pittsburgh, PA 15213, USA; Department of Pathology, University of Pittsburgh, Pittsburgh, PA 15213, USA; UPMC Hillman Cancer Center, Pittsburgh, PA 15213, USA

## Abstract

Alterations in the tumor suppressor *ATRX* are recurrently observed in mesenchymal neoplasms. ATRX has multiple epigenetic functions including heterochromatin formation and maintenance and regulation of transcription through modulation of chromatin accessibility. Here, we show in murine mesenchymal progenitor cells (MPCs) that *Atrx* deficiency aberrantly activated mesenchymal differentiation programs. This includes adipogenic pathways where ATRX loss induced expression of adipogenic transcription factors and enhanced adipogenic differentiation in response to differentiation stimuli. These changes are linked to loss of heterochromatin near mesenchymal lineage genes together with increased chromatin accessibility and gains of active chromatin marks. We additionally observed depletion of H3K9me3 at transposable elements, which are derepressed including near mesenchymal genes where they could serve as regulatory elements. Finally, we demonstrated that loss of ATRX in a mesenchymal malignancy, undifferentiated pleomorphic sarcoma, results in similar epigenetic disruption and de-repression of transposable elements. Together, our results reveal a role for ATRX in maintaining epigenetic states and transcriptional repression in mesenchymal progenitors and tumor cells and in preventing aberrant differentiation in the progenitor context.

## Graphical Abstract

**Graphical Abstract:**
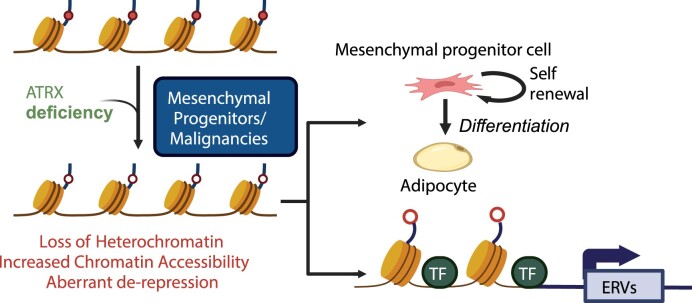

## Introduction

Alpha thalassemia/mental retardation syndrome X-linked (ATRX) belongs to the SWI/SNF family of ATP-dependent chromatin remodeling proteins (1). Germline mutations in ATRX cause cognitive impairment as part of the alpha-thalassemia (ATR-X) syndrome, which is accompanied by disturbances of DNA methylation (2), highlighting the function of ATRX in development (3). Somatic alterations in *ATRX* occur in cancers, such as sarcomas (4).

ATRX is an important epigenetic regulator, functioning as a chromatin remodeler and promoting the formation and maintenance of heterochromatin (5). For example, ATRX cooperates with the H3K9 methyltransferase SETDB1 to establish and maintain heterochromatin, including at retrotransposons (5). ATRX also binds to H3K9me3 via its ADD domain to directly target it to heterochromatin, which may be important for its role in maintaining H3K9me3 domains (5). ATRX also binds to DAXX, functioning as a histone chaperone depositing H3.3 into telomeric regions (6,7).

Loss of function genetic alterations in *ATRX* are highly recurrent in several cancers including gliomas, pancreatic neuroendocrine tumors, and multiple sarcoma subtypes (8,9). Within sarcomas, *ATRX* is altered in more than 10 percent of undifferentiated pleomorphic sarcoma, leiomyosarcoma, myxofibrosarcoma, perivascular epithelioid tumors, pleomorphic liposarcoma and angiosarcoma. In uterine leiomyosarcoma (ULMS), the frequency of *ATRX* alterations is approximately one third of cases (8). Consistent with these genetic studies, loss of ATRX expression occurs in 20–30% of undifferentiated pleomorphic sarcoma (UPS) and leiomyosarcomas (10,11). While the role of ATRX deficiency in the alternative lengthening of telomeres pathway is well described in sarcomas and other ATRX deficient cancers, the chromatin-specific consequences of ATRX loss are not fully understood in the context of disease mechanisms (12,13).

Sarcomas are mesenchymal neoplasms occurring in connective tissue such as fat, bone, cartilage and muscle (14). It has been proposed that sarcomagenesis occurs, at least in part, through aberrant differentiation of mesenchymal progenitor cells (MPCs), which has been modeled by introducing sarcoma-relevant driver alterations into MPCs (15–19). MPCs are multipotent cells that are able to self-renew and undergo differentiation (20) making them well-suited to study perturbations in epigenetic states, which are a key determinants of cell fate and lineage commitment (21–26). For example, sarcoma-associated mutations in histone genes, which alter histone posttranslational modifications, are sufficient to induce sarcomagenesis when expressed in MPCs (27,28). Given the high frequency of *ATRX* alterations in soft tissue sarcoma, we sought to investigate the effect of ATRX deficiency on chromatin and chromatin-dependent processes in the mesenchymal context.

Our findings demonstrate that deletion of ATRX leads to abnormal differentiation in MPCs, which is associated with perturbations in the profiles of histone post-translational modifications and chromatin accessibility in specific regions, and accompanied by the activation of transposable elements. Similar epigenetic effects were observed in an ATRX deficiency patient-derived sarcoma cell compared to a wildtype isogenic control. Our results suggest an important role for the epigenetic regulatory functions of ATRX in the mesenchymal lineage.

## Materials and methods

### Cell culture

C3H/10T1/2 (CCL-226) cells (MPCs) were obtained from ATCC. Except where otherwise stated for specific experiments, cells were cultured in DMEM (Corning, 10-013-CV) plus 10% FBS (ATLANTA biologicals, S11150, heat inactive) with 1% penicillin/streptomycin (Gibco, 15140-122) at 37°C culture condition with 5% CO_2_. The parental cell line was tested for mycoplasma and authenticated by ATCC. The undifferentiated pleomorphic sarcoma cell line (UPS, 3672–3) was developed in the Singer lab (*TP53* deficient with retained RB1 expression) (29). The cells were cultured in DMEM:F12 (1:1) (Gibco, 11330-032) plus 10% FBS (ATLANTA biologicals, S11150) and l-glutamine (Gibco, 25030-081) with 1% penicillin/streptomycin (Gibco, 15140-122) at 37°C with 5% CO_2_. The parental cell line was tested for mycoplasma.

### CRISPR-Cas9 cloning

The vector for CRISPR-cas9 pSpCas9(BB)-2A-GFP (PX458) (Addgene, #48138) was obtained from Addgene. Mouse *Atrx* sgRNAs were designed using Benchling (https://www.benchling.com/). Target sequences: sg5: 5′ TGGCCGTAAAAGTTCTGGGG-3′, sg6: 5′-CTACTGGACTTGGTGACTGC-3′. The human *ATRX* sgRNA was designed using Benchling; Target sequence: sg1: 5′ ACTATGCAGAGCTTGCCAAA-3′. The control group was generated using an empty vector without sgRNA but carrying Cas9. The vector was digested by Bbsl (NEB, R0539S) and dephosphorylated by calf intestinal alkaline phosphatase (NEB, M0290). The oligos were annealed with T4 ligation buffer (NEB, B0202S) with T4 PNK (NEB, M0201L) enzyme. The ligation step was performed using Quickligation buffer (NEB, B2200S) and Quick Ligase (NEB, M2200L). The ligation products were expressed in Stbl3 competent cells (Thermofisher, C737303).

### Transient transfection

Two micrograms of plasmid products carrying sgRNAs with Cas9 were transfected using Lipo2000 (Thermofisher, 11668019) into 0.7 × 10^4^ MPCs cells or 1 × 10^5^ UPS cells. The transfection procedure was according to the manufacturer's instructions. Cells were harvested 48 h after transfection for sorting and sorted into 96 well plates using BD FACSAria II Cell Sorter based on GFP signal.

### Proliferation assay

Fifty *Atrx* WT and *Atrx* KO cells were grown per well in 96-well plates (Costar, 3917) for 5 days. Proliferation was evaluated using the CellTiter-Glo Luminescent Cell Viability Assay kit (Promega, G7572), following the manufacturer's instruction. The luminescence was read immediately in 96-well plate reader (BioTek, Synergy hybrid H4 Reader).

### Colony formation

100 C3H/10T1/2 cells (WT and KO clones) were seeded in 10 cm dishes, cultured in α-MEM (Corning, 10-009-CV) with 20% FBS (ATLANTA biologicals, S11150), with 1% penicillin/streptomycin (Gibco, 15140–122). Cultures were incubated at 37-degree Celsius with 5% CO_2_ for 10–14 days. When visible colonies formed, colonies were washed once with PBS. Cells were stained with 0.5% crystal violet (SIGMA-ALDRICH, C3886) in 4% formaldehyde (Fisher chemical, F79-500) for 1 h at RT. After removing the staining buffer, cells were washed with ddH_2_O for 3–5 min to remove the background staining and then digitally imaged (Epson Perfection V800 photo). Colonies were manually counted. A group of cells had to contain at least 50 cells to be considered a colony.

### Adipogenic differentiation

C3H/10T1/2 were differentiated into mature adipocytes following treatment with insulin, dexamethasone, troglitazone, and methylisobutylxanthine per an established protocol (30). 1 × 10^5^ C3H/10T1/2 were seeded in 24-well plates such they were fully confluent. Adipocyte differentiation medium (30) consisting of DMEM (Corning, 10-013-CV) with 10% heat inactive FBS (ATLANTA biologicals, S11150), 0.5 mM isobutyl methylxanthine (Sigma, I7018), 1 μM dexamethasone (sigma, D4902), 5 μg/ml insulin (Sigma, I9278), 5 μM troglitazone (Sigma, T2573) was used to replace the media the following day. After 2 days, the media was changed every 2 days with insulin and FBS the only additive to the DMEM. Differentiation was assessed by Oil Red O after day 7.

### Oil Red O staining

Oil Red O staining was performed as previously described (30). 0.5% w/v Oil Red O stock (Sigma, O0625) in 100% isopropanol (Fisher chemical, A416SK-4) was prepared fresh. A working solution was prepared by mixing contained 6 ml of stock solution and 4 ml of H_2_O, followed by filtering (0.45 μM, Pall Corporation syringe filter, PN-4614) to remove precipitate. Cells were washed by PBS. Cells were fixed with 4% formaldehyde in PBS for 2 min at room temperature, followed by washing with H_2_O. Next, 220 μl Oil Red O working solution was added to the wells and incubated cells for 1 h at room temperature. Then cells were washed twice with 0.5% isopropanol to remove background staining. Plates were digitally imaged (Epson Perfection V800 photo) before the Oil Red O was solubilized by adding 650 μl of 100% isopropanol to the stained cells, which were incubated for 20 min on a shaker. The eluate was transferred into a 96-well plate (Corning, 3361) and the absorbance measured at 500 nm with 96-well plate reader (BioTek, Synergy hybrid H4 Reader).

### Chondrocyte differentiation

C3H/10T1/2 were differentiated into chondrocyte following treatment with insulin, sodium selenite, transferrin, dexamethasone and rhBMP-2 as previous described (27). 1 × 10^5^ C3H/10T1/2 were seeded per well in 24-well plates such that they were fully confluent. Chondrocyte differentiation medium consisting of DMEM (Corning, 10-013-CV) with 1% heat inactive FBS (ATLANTA biologicals, S11150), 10 μg/ml insulin (Sigma, I9278), 0.03 μM Sodium Selenite (Sigma, S5261), 0.01 mg/ml Transferrin (Sigma, T8158), 10 nM Dexamethasone (Sigma, D4902), 0.1 μg/ml rhBMP-2 (Peprotech, 120-02) was used to replace the media the following day. After 2 days, the media was changed every 2 days. Alcian blue staining was performed after 9 days of differentiation.

### Alcian blue staining

Cells were washed by PBS twice and then fixed with 4% formaldehyde for 2 min at room temperature. Formaldehyde was removed and cells were washed with ddH_2_O. 1% Alcian blue 8GX (Sigma, 66011) was used to stain cells for 1h at room temperature. After staining, cells were washed with acetic acid twice and then quickly with ddH_2_O. The plate was air dried and then digitally imaged (Epson Perfection V800 photo). For quantification, 1% SDS was added to the well and the plate was placed on a shaker overnight at room temperature. One hundred microliters of the eluate was transferred to a well of a 96-well plate (Corning, 3361) and the absorbance was measured at 605 nm (BioTek, Synergy hybrid H4 Reader).

### Immunofluorescence

800 C3H/10T1/2 cells were seeded per well in a 96 well plate (glass bottom culture plates, MatTek, PBK96G-1.5-5-F) and kept at 37-degree Celcius for 24 h. Cells were fixed with 4% formaldehyde in PBS for 10 min at room temperature. Cells were permeabilized with 0.2% digitonin (EMD Millipore, 300410) in PBS (Corning, 21-040-CV) for 10 min at room temperature. Next, the cells were incubated in 3% BSA (in PBS, Sigma, #9418) for blocking for 1 h at room temperature followed by incubation in primary antibody (ATRX, Santa Cruz, sc-15408, 1:200) at 4°C overnight, followed by secondary antibody (1:600, Invitrogen, A32754) incubation at room temperature for 1 h. Cells were washed three times with PBS for 5 min followed by DAPI staining (2 μg/ml in PBS, Sigma, D9564) for 5 min at room temperature. Mounting media (Vectashield, H-1000) was added immediately after DAPI staining. Cells were imaged using Widefield Microscope CellDiscoverer7 (CD7) automated widefield high-throughput system (Zeiss). Images were processed with ImageJ software (http://rsb.info.nih.gov/ij/). For the ATRX antibody, the ImageJ Brightness/Contrast was set as 30/112. For DAPI, the Brightness/Contrast was set as 37/123. All images were processed with the same parameter settings for each antibody.

### Protein isolation and western blot

Cell pellets were lysed in lysis buffer NETN (20 mM Tris (pH 7.5), 1 mM EDTA, 150 mM NaCl, 0.5% NP-40, protease inhibitor tablet (Roche), 0.5 mM DTT). Samples were incubated in the cold room for 30 min followed centrifugation at 4°C with maximum speed for 10 min. The supernatant was collected and the concentration determined by BCA quantification (Pierce BCA protein Assay kit, Cat.23225, Thermo scientific) to allow normalization between samples for western blotting. Samples were mixed with Laemmli Sample Buffer (4×) (containing 1.0 M Tris-pH 6.8, 8% SDS, glycerol, β-mercaptoethanol (10%), bromophenol blue) for boiling at 100°C for 10 min. The samples were then separated by SDS-PAGE and analyzed by standard immunoblotting using running buffer from Invitrogen (NuPAGE™ Tris-acetate SDS running buffer (20×), LA0041) and transfer buffer from Thermofisher (NUPAGE transfer buffer, NP00061). The blotting processes was performed as previous described (27). Antibodies used for Western blot are the following: ATRX (Santa Cruz, sc-15408, 1:800), FABP4 (R&D, AF1443, 1:5000), C/EBPα (CST, #2295, 1:1000), PPARγ (CST, #2430), β-actin (Abcam, ab8224), GAPDH (Abcam, ab8245, 1:1000).

### Reverse transcriptase quantitative PCR (RT-qPCR)

For RT-qPCR, RNA was prepared with RNeasy Mini kits (Qiagen, 74104). The RNAs concentration was determined using a Nanodrop (Spectrophotometer, ND-1000). cDNA was prepared published procedures (27). qPCRs were performed using SYBR green PCR master mix (Applied Biosystems, 4367659). The detailed steps are following previous described (27). The endogenous control gene was 18S. The sequences of primers are as following: mouse-Etv1: F:5′-GTTTGTTCCAGACTATCAGGCTG-3′, R: 5′-GGGCTGTGGGGTTCTTTCTT-3′. mouse-18s: F:5′-GTAACCCGTTGAACCCCATT-3′, R: 5′-CCATCCAATCGGTAGTAGCG-3′. The statistical analysis was performed using a one-sample, two-sided *t-*test.

### UPS cell line drug treatment and dose–response calculations

2000 UPS cells were seeded in each well of a 96-well plates (Costar, 3917) and grown for 24 h. The media was replaced with fresh media containing different concentrations of tegavivint (Selleckchem, S0733) or vehicle (DMSO) and returned to the incubator for 72 h. Viability was then evaluated using the CellTiter-Glo Luminescent Cell Viability Assay kit (Promega, G7572), following the manufacturer's instruction. The luminescence was read immediately in a 96-well plate reader (BioTek, Synergy hybrid H4 Reader). The IC_50_ was calculated by GraphPad Prism 8.4.2 using curve fitting for log(inhibitor) versus response with variable slope (four parameters) with the bottom constraint set as 0.

### PolyA-RNA-seq and data analysis

Approximately 1 million C3H/10T1/2 cells or UPS cells were collected from 10 cm dishes. RNA extraction, polyA selection, library preparation, and RNA sequencing were performed at the MSKCC integrated Genomics Operation. An average of 30–40 million paired reads was generated per sample. We used the log_2_foldchange >1 or <–1 with *P*_adj_ <0.05 as the threshold for significant changed genes. Quality control of FASTQ files was performed using the Rfastp R Bioconductor package (v0.1.2). For 10T cells, full genome sequence and transcript coordinates for the mm10 UCSC genomes and gene models were retrieved from the R Bioconductor packages BSgenome.Mmusculus.UCSC.mm10 (v1.4.0) and TxDb.Mmusculus.UCSC.mm10.knownGene (v3.4.0), while for human UPS cells, the full genome sequence and transcript coordinates for the hg38 UCSC genomes and gene models were retrieved from the R Bioconductor packages BSgenome.Hsapiens.UCSC.hg38 (v1.4.1) and TxDb.Hsapiens.UCSC.hg38.knownGene (v3.4.0).Transcript abundance was determined from FASTQ files using Salmon (v0.8.1) (31), and transcript counts and TPM values were imported into R with the tximport R Bioconductor package (v1.8.0) (32). Differential gene expression was performed with the DESeq2 R Bioconductor package (v1.20.0) (33). For plots comparing the read counts of genes between samples, counts were normalized by dividing the raw read counts by the size factors for each sample as determined by DESeq2. To perform differential expression of TEs in the human and published mouse (GEO accession GSE167537) UPS poly-A selected RNA-seq datasets, the SQuIRE pipeline was used for alignment, counting, and calling of differential TE expression (v0.9.9.9a-beta: https://github.com/wyang17/SQuIRE).

### rRNA-depletion RNA-seq and data analysis

Approximately 1 million cells were collected from 10 cm dishes. RNA extraction, rRNA depletion, library preparation, and RNA sequencing were performed by Novogene. An average of 30 million paired reads was generated per sample. Quantification of rRNA-depleted RNAseq reads over individual TE loci was performed using either the SQuIRE pipeline (v0.9.9.9a-beta: https://github.com/wyang17/SQuIRE) (34) or TE local (v1.1.1: https://github.com/mhammell-laboratory/TElocal). The SQuIRE pipeline was used for alignment, counting, and calling of differential TEs. When using TElocal, reads were aligned with STAR (v2.27.10a) setting the ‘—winAnchorMultimapNmax’ and ‘—outFilterMultimapNmax’ arguments to 100 (35), followed by counting with the TElocal function and differential abundance calculated with DESeq2 (v1.32.0). Reads were aligned to the mm10 genome sequence from the BSgenome.Mmusculus.UCSC.mm10 R Bioconductor package (v1.4.0).

### CUT&RUN

0.5 million cells from each line were collected. The detailed protocol for CUT&RUN for histone marks (H3K4me3, H3K27ac, H3K9me3, H3K27me3) and H3.3 was followed as previous described (36) with the following modifications: final concentration of digitonin: 0.05%. Antibodies for CUT&RUN are following: H3.3 (Active Motif, 91191), H3K9me3 (Abcam, ab8898), H3K4me3 (Active Motif, 39159), H3K27me3 (Cell Signaling Technologies, #9733), H3K27ac (Active Motif, 39133), Rabbit IgG (Diagenode, C15410206). For ATRX CUT&RUN experiment, 1 million cells were fixed using 0.1% PFA in PBS for 1 minute at room temperature, followed by nuclei extraction with NE buffer (20 mM HEPES (pH7.9), 10 mM KCl, 0.1% Triton X-100, 20% Glycerol, 1 mM MnCl2, 1 × cOmplete Mini-Tablet (1 tablet), 0.5 mM spermidine) according to the protocol from EpiCypher (v2.0, https://www.epicypher.com/resources/protocols/cutana-cut-and-run-protocol/). The final concentration of digitonin buffer for ATRX CUT&RUN was 0.01%. For each sample, 2 μg ATRX antibody (Abcam, ab97508) was added. Libraries were prepared using the NEB Ultra II DNA library prep kit. Samples were PCR amplified for 14 cycles and pooled libraries were sequenced in the genomics core at the Rockefeller University.

### CUT&RUN alignment and differential peak analysis

Quality control of FASTQ files was performed using the Rfastp R Bioconductor package (v0.1.2). CUT&RUN reads were aligned using the Rsubread R Bioconductor package (v1.30.6), and predicted fragment lengths were calculated by the ChIPQC R Bioconductor package (v1.16.2) (37,38). For 10T cells, the full mm10 genome sequence was retrieved from the R Bioconductor package BSgenome.Mmusculus.UCSC.mm10 (v1.4.0), and for the human UPS cells the full hg38 genome sequence was retrieved from the R Bioconductor package BSgenome.Hsapiens.UCSC.hg38 (v1.4.1). Normalized, fragment-extended signal bigWigs were created using the rtracklayer R Bioconductor package (v1.40.6) (39). Range-based heatmaps showing signal over genomic regions were generated using the profileplyr R Bioconductor package (v1.8.1) (40). Any regions included in the ENCODE blacklisted regions of the genome were excluded from all region-specific analyses (41). Bedgraph files generated with the deepTools package (v3.5.1) (42) were used for peak calling with SEACR (v1.3) (43). The SEACR ‘stringent’ mode was used for H3K4me3, H3K27ac and ATRX and the ‘relaxed’ mode was used for H3K9me3 and H3.3. For all CUT&RUN samples, peaks were called using IgG as the control sample.

Differential enrichment of signal within peaks was performed by counting the overlap of reads over a high confidence consensus peak set. This was obtained by reducing all replicates within all conditions to one peak set, and then keeping the peaks that overlap at least two out of the three replicates for any condition. Reads overlapping these peaks were counted for each replicate using the summarizeOverlaps function from the GenomicAlignments R Bioconductor package (v1.28.0) (44). Differential peak enrichment between *Atrx* WT and *Atrx* KO replicates was then calculated using the DESeq2 R Bioconductor package (v1.32.0). To find differential enrichment in broad genomic regions for the H3K9me3 samples, reads were counted in 20 kb bins across the entire genome, and DESeq2 was used to find bins with enriched signal in the *Atrx* KO clones. Only bins in the top 10 percent in terms of read count were used for this analysis. The union of these differential bins and the differential SEACR peak regions was determined and used for downstream analysis of regions with enriched H3K9me3 signal in the *Atrx* KO clones.

Quantification of CUT&RUN signal over repeats at the individual locus and sub family level was performed with SQuIRE. The counts were transformed with rlog from the DESeq2 R Bioconductor package (v1.32.0), and these values were plotted on a heatmap using the ComplexHeatmap R Bioconductor package (v2.6.2) (45). To assess ARTX CUT&RUN signal in telomeres, FASTQ files were aligned to a DNA sequence of 150 conserved telomere repeats (TTAGGG) using the R Bioconductor package Rbowtie2 (v1.12.0) (46,47). The z-scores of the log2(reads per million) were plotted on a heatmap using the ComplexHeatmap R Bioconductor package.

For quantification of ATRX CUT&RUN signal over genes, genes were divided into groups based on their expression levels. TPM values from the *Atrx* WT RNAseq dataset were calculated by Salmon and were split into five quantiles. The genomic location of each gene was then retrieved from the R Bioconductor package TxDb.Mmusculus.UCSC.mm10.knownGene (v3.10.0). IgG normalized bigwig signal for the ATRX CUT&RUN samples was obtained using the deepTools (v3.5.1) ‘bigwigCompare’ function, with the ‘operation’ argument set to ‘log_2_’. Signal over the gene regions (separated by RNAseq quantile) was computed with the deepTools computeMatrix function. The signal was visualized with the ggplot2 R package (v3.3.6).

### Omni-ATAC-seq

The samples were prepared according to the methods previous described (48–50) with minor modifications. 50 000 C3H/10T1/2 cells or UPS cells were collected for Omni-ATAC-seq. Amplification was performed with NEBNext High-Fidelity 2 × PCR Master Mix (NEB, M0541s) and Nextera PCR Primers (8 cycles). The sequences of PCR primers with index adapters are listed in the Supplementary Table S38. The library pool was sequenced by Rockefeller University genomic core using a NextSeq High Output platform with 75 bp paired-end reads in duplicates. An average of 30–40 million paired reads was generated per sample.

### ATAC-seq alignment and differential peak analysis

Quality control of FASTQ files was performed using the Rfastp R Bioconductor package (v0.1.2). ATAC-seq FASTQs were aligned to the mm10 (for 10T cells) or hg38 (human UPS cells) genomes from the Bsgenome.Mmusculus.UCSC.mm10 (v1.4.0) or the BSgenome.Hsapiens.UCSC.hg38 (v1.4.1) Bioconductor packages, respectively, using Rsubread's (v1.30.6) align method in paired-end mode with fragments between 1 and 5000 bp considered properly paired (38). Normalized, fragment signal bigWigs were created using the rtracklayer R Bioconductor package (v1.40.6) (39). Peak calls for each replicate were made with MACS2 software in BAMPE mode (51).

Differential enrichment of signal within peaks was performed by counting the overlap of reads over a high confidence consensus peak set. This was obtained by reducing all replicates in all conditions to one peak set, and then keeping all peaks that overlap at least two out of the three replicates for any condition. Reads overlapping these peaks were counted for each replicate using the summarizeOverlaps function from the GenomicAlignments R Bioconductor package (v1.28.0) (44). Differential peak enrichment between *Atrx* WT and *Atrx* KO replicates was then calculated using the DESeq2 R Bioconductor package (v1.28.1).

### Motif analysis

Motif analysis for ATAC-seq and ATRX CUT&RUN was performed with the ‘meme-chip’ function from the MEME suite (v5.4.1). The motif database was downloaded from MEME (https://meme-suite.org/meme/doc/download.html) and the ‘jolma2013.meme’, ‘JASPAR2022_CORE_vertebrates_non-redundant_v2.meme’, and ‘uniprobe_mouse.meme’ were used in the meme-chip function. The following parameters were also set: -ccut 200, -dna -order 2, -minw 6, -maxw 15, -meme-mod zoops, -meme-nmotifs 3, -meme-searchsize 100000, -streme-pvt 0.05, -streme-totallength 4000000, -centrimo-score 5.0, and -centrimo-ethresh 10.0.

### Downstream peak analysis (CUT&RUN and ATAC-seq)

Peaks for both CUT&RUN and ATAC-seq were annotated with nearby genes using the rGREAT R Bioconductor package (v1.24.0) or with the closest gene and type of genomic region (e.g. promoter, intergenic, etc.) using the ChIPseeker R Bioconductor package (v1.28.3) (52–54). The closest gene is defined as the gene for which the transcription start site is closest to the peak, and rGREAT defines a regulatory region around each gene and then annotates any peak in that region to that gene. For rGREAT annotation, the default settings were used to define regulatory regions of genes, which was 5 kb upstream and 1 kb downstream of each gene, and then extended a maximum of 1 Mb to the regulatory region of the next gene. Gene ontology analysis of genes associated with differential peaks (using the Biological Processes gene lists) was performed with the clusterProfiler R Bioconductor package (v4.0.5) (55). Overlaps of differential peaks with other peak sets was done with the findOverlapsOfPeaks function from the ChIPpeakAnno R Bioconductor package (v3.26.4) (56). Range heatmaps of CUT&RUN and ATAC-seq signal were made with the profileplyr R Bioconductor package (57). For overlap of signal and peaks with enhancers, a peak file with enhancers from 10T cells was downloaded from the EnhancerAtlas 2.0 (http://www.enhanceratlas.org/). The downloaded peaks were converted from mm9 to mm10 using the ‘liftOver’ function from rtracklayer R Bioconductor package and the ‘mm9ToMm10.over.chain’ file downloaded from UCSC.

### ChromHMM analysis

Genome-wide ChromHMM (v1.23) models were generated using CUT&RUN signal for H3K4me3, H3K27ac, H3K27me3, H3K9me3, with IgG samples used as controls. Only the CUT&RUN samples from the *Atrx* WT cells were used to generate the models. Specifically, the BinarizeBam function was performed with default settings (mm10 genome), and this output was used in the LearnModel function. To determine the optimal number of states, we employed a previously published method to calculate the ratio of the ‘between sum of squares’ over the ‘total sum of squares’ of kmeans clustered emissions probabilities from all states of all models, considering 2–16 states (58). The value of K at which the sum of squares ratio crossed 95% of the maximum was the 10 State model. To determine overlap of ChromHMM states with repeats, repeats from RepeatMasker were downloaded from UCSC (downloaded on 4 February 2022). To compare levels of overlap between states, the number of repeats that overlap a state was divided by the total number of regions in that state. A *z*-score of this value across all states was then plotted in a heatmap using the ComplexHeatmap R Bioconductor package (v2.6.2). A similar strategy was employed to measure ATRX peak overlap with ChromHMM states. H3K9me3 signal in specific states was quantified and visualized with a ranged heatmap using the profileplyr R Bioconductor package.

### Statistics

Statistical analysis for proliferation, colony formation and qPCR were performed as indicated in the figure legends using paired or unpaired, two-tailed *t-test, or one-way ANOVA*. The analysis was not blinded.

## Results

### Loss of ATRX dysregulates transcriptional programs in mesenchymal progenitors

**Figure 1.**
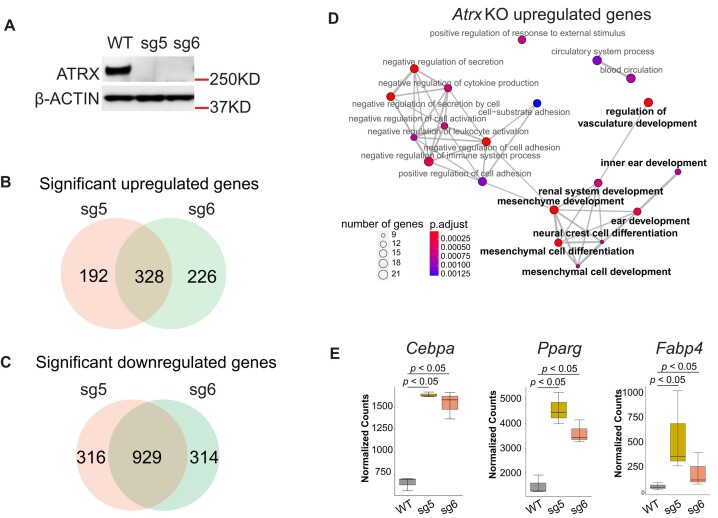
ATRX deficiency alters the transcriptome. (**A**) Immunoblot demonstrating ATRX protein loss in MPC lines. β-ACTIN acts as loading control. (**B**) The intersection of significantly upregulated genes (log_2_foldchange > 1, *P*_adj_< 0.05) in both *Atrx* KO clones based on polyA-RNA-seq datasets. (**C**) The intersection of significantly downregulated genes (log_2_foldchange < –1, *P*_adj_ value < 0.05) in both *Atrx* KO clones based on polyA-RNA seq datasets. (**D**) Dot plot of gene ontology (GO) (biological process) analysis for significant upregulated genes from (B). The size of nodes indicates the numbers of genes. The color gradient indicates the *P_adj_* value. Bold type indicates gene ontology terms which are associated with development. (**E**) Boxplot depicting mRNA levels from the RNA-seq dataset. The y-axis indicates RNA-seq read counts normalized by DESeq2. The *P* value (*P*< 0.05) was determined by DESeq2.

It is well established that the chromatin landscape has a key role in establishing patterns of gene expression, and since ATRX acts as chromatin regulator we hypothesized that ATRX deficiency would lead to changes in transcription. Given that ATRX loss frequently occurs in mesenchymal malignancies, we established *Atrx* KO lines using C3H/10T1/2 cells, a mesenchymal progenitor cell line, using CRISPR-cas9 with two independent sgRNAs targeting the *Atrx* gene (Supplementary Figure S1A). One clone from each guide and an isogenic WT control were selected for further study (Figure 1A, Supplementary Figure S1B). ATRX deletion was confirmed by immunofluorescence and immunoblotting (Figure 1A, Supplementary Figure S1C). Under normal culture conditions without differentiation cues, transcriptomic profiling of *Atrx* KO versus WT cells was performed using bulk RNA-seq. Compared to *Atrx* WT, the *Atrx* KO MPC lines displayed a marked transcriptional dysregulation, with significant gains and losses of gene expression (Supplementary Figure S2A,B). Focusing on genes that significantly change expression (*p_adj_ <*0.05) by an absolute magnitude of at least 2-fold in both *Atrx* KO clones when compared to WT, we identified 328 upregulated and 929 downregulated genes (Figure 1B, C, Supplementary Table S1, S2). Given the role of ATRX in establishing and maintaining transcriptionally silenced regions of heterochromatin, we focused on the upregulated gene set. Gene ontology (GO) analysis of the upregulated genes showed an enrichment of gene sets associated with development and differentiation programs, including mesenchymal programs (Figure 1D, Supplementary Table S3).

In the significantly upregulated gene sets, we found that the expression of key adipogenic pathway regulators were increased with *Atrx* deficiency, including the adipogenic transcription factors *Pparγ* and *Cebpa* and the lineage-specific marker *Fabp4*. All three were significantly (log_2_foldchange > 1 & *P*_adj_ < 0.05) upregulated in *Atrx* KO MPCs (Figure 1E, Supplementary Table S4), even in the absence of adipogenic differentiation factors. Under the same conditions, we observed significantly decreased expression of mesenchymal stemness markers, *Etv1* (27) and *Cd34* (59), in both *Atrx* KO lines (Supplementary Figure S2C). The GO terms associated with downregulated genes were not enriched for pathways relevant for cell lineage differentiation and development (Supplementary Figure S2D, Supplementary Table S5).

### ATRX deficiency promotes mesenchymal lineage differentiation and attenuates progenitor properties

**Figure 2.**
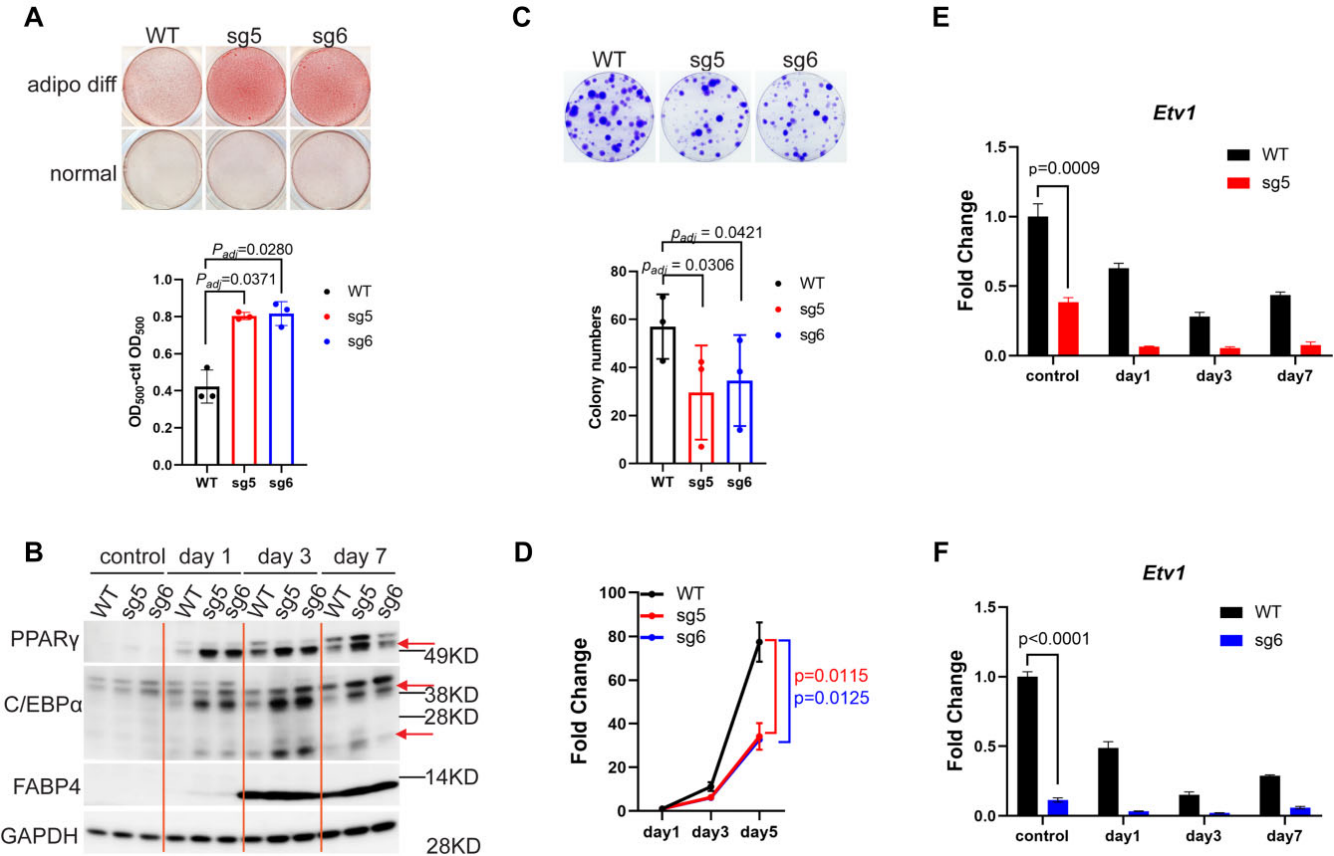
ATRX deficiency promotes differentiation. (**A**) Oil Red O (ORO) staining in adipogenic differentiation experiments. adipo diff: adipogenic media treatment. normal: normal culture media treatment. The y-axis shows the optical density (OD) of the solubilized ORO after adipocyte-specific staining minus the OD value from the normal media control (clt). Data from three biological replicates are plotted with each point representing individual value from each replicate. The *p*.adj value was calculated by a post hoc comparison following a paired one-way ANOVA analysis (**B**) Western blot of adipocyte markers. The ‘control’ indicates cells grown in the normal media culture at the end of the experiment (Day 7). Days 1, 3 and 7 indicate the duration of adipogenic media treatment. The red arrows indicate the protein bands of interest. Two isoforms of C/EBPα are shown. (**C**) Colony formation assays. The bar plot shows the colony formation from three biological replicates normalized to WT. Cell groups with at least 50 cells were classified as a colony. For each replicate, the percentage of colony formation was normalized to the WT group. The error bars were calculated from three biological replicates. The *P* value was determined by paired one-way ANOVA (WT versus sg5, WT versus sg6). (**D**) MPC proliferation assay. Relative numbers of viable cells were determined using an ATPase assay on day 1, day 3 and day 5 after seeding cells. The OD value of luminescence was normalized with day 1 for each group. Three biological replicates were performed. *P* value was calculated by paired *t-test* (two-tailed) for day 5 data. (**E**, **F**) RT-qPCR experiments for *Etv1* gene expression in two knockout lines Corresponding to the data in panel (B). *P* value was calculated using one-sample, two-sided *t-*test to compare the mean values for sgRNA samples and WT. The error bars indicate the standard variance.

The de-repression of developmental and differentiation pathways in *Atrx* KO cells suggests that ATRX may have an important role in restricting differentiation in MPCs. Given the marked upregulation of adipogenic transcription factors and lineage markers in *Atrx* KO lines, we hypothesized that ATRX loss would sensitize MPCs to adipogenic differentiation cues. *Atrx* WT and KO MPCs were treated with adipogenic media to induce differentiation and the degree of differentiation was quantified by staining by Oil Red O (ORO) (30). Compared to WT MPCs, the *Atrx* KO lines nearly doubled ORO staining intensity after 7 days (*P*< 0.05), indicating that ATRX loss promotes adipogenic differentiation (Figure 2A). Over the course of the 7-day differentiation treatment, we measured protein expression of PPARγ, C/EBPα and FABP4 (Figure 2B). The levels of C/EBP and PPARγ were more highly induced beginning on day 1 of treatment in *Atrx* KO lines compared to WT control whereas the FABP4, a marker for late differentiation, was equally and strongly induced in both settings beginning on day 3. To investigate if the regulatory role of ATRX in mesenchymal differentiation is specific to the adipocyte lineage, we also tested the differentiation of *Atrx* KO versus WT MPCs following treatment with a chondrocyte differentiation cocktail. The H3K36M oncohistone mutation was used as a negative control (27). Compared to WT MPCs, the H3K36M-expressing negative control led to impaired differentiation (Supplementary Figure S3). In addition, both ATRX deficient clones demonstrated reduced differentiation into chondrocytes (Supplementary Figure S3). These data suggest that ATRX loss perturbs the lineage commitment of mesenchymal progenitor cells resulting in selective linage promotion or impairment.

To assess if *Atrx* KO reduced the stemness properties of mesenchymal progenitors, we performed assays for colony formation (Figure 2C) and proliferation (Figure 2D). The two *Atrx* KO lines had reduced colony formation capacity and proliferation compared to *Atrx* WT (*P*< 0.05). We also compared mRNA levels of *Etv1*, a mesenchymal stemness marker, between *Atrx* KO and WT MPCs (Figure 2E, F). The two knock out lines expressed lower levels of *Etv1* during the differentiation time course, including at Day 0, which is consistent with the RNA-seq findings, suggesting that *Etv1* is suppressed at baseline in *Atrx* KO MPCs. These results demonstrate that *Atrx* KO reduces the stem-like properties of mesenchymal progenitors.

### Atrx KO reduces H3K9me3-marked heterochromatin associated with transcriptionally repressed linage commitment genes

Histone post-translational modifications contribute to defining the chromatin states, which in turn impact gene expression (60,61). Given that histone post-translational modifications are regulated in part by ATRX, we investigated how ATRX deficiency impacted chromatin states in the MPC model. ATRX, together with DAXX, deposits the histone variant H3.3 at specific genomic regions including telomeres (6,7,62). To confirm loss of ATRX epigenetic function in the knockout cell lines, we performed CUT&RUN (Cleavage Under Targets & Release Using Nuclease) (36) for the histone variant H3.3 in order to compare H3.3 deposition in *Atrx* KO versus WT MPCs. Analysis of three biologic replicates indicated that there were consistent changes in H3.3 localization with gains at some genomic loci and losses at others (Supplementary Figure S4A) including significant depletion at telomeres (Supplementary Figure S4B), which is consistent with the previous reports (63). Notably, H3.3 localization is also known to change over the course of development (7).

Another known function of ATRX is to establish and maintain locus-specific heterochromatin though recruitment of H3K9 methyltransferases (5). Using CUT&RUN, we next analyzed how the heterochromatin-associated histone mark, H3K9me3, was altered in *Atrx* KO MPCs. H3K9me3 was significantly changed at specific regions with 4272 gained peaks and 4738 lost peaks, demonstrating that the ATRX deficiency has effects on heterochromatin in our model (Supplementary Figure S4C). Applying GO analysis to genes that are near H3K9me3 differential peaks, we found that many of the top enriched gene sets for regions that had increased or decreased H3K9me3 signal in both *Atrx* KO lines were related to development (Supplementary Figure S4D,E; Supplementary Table S6, S7).

**Figure 3.**
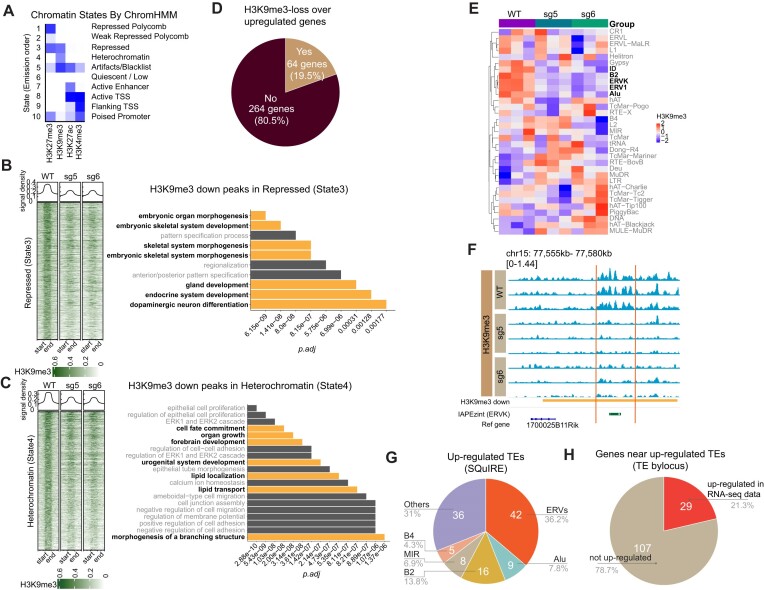
ATRX deficiency reduces H3K9me3 near developmental genes and ERVs in mesenchymal progenitor cells. (**A**) Chromatin states defined by enrichment of histone modifications (H3K4me3, H3K9me3, H3K27ac and H3K27me3) using ChromHMM. The heatmap shows the emission probabilities of histone modifications in chromatin states. The ten different states. (**B**) The range-based heatmap shows the H3K9me3 signal in repressed regions (State 3) (left) and (**C**) heterochromatin regions (State 4) (left). All peaks are similarly scaled, where ‘start’ and ‘end’ indicate the start and end of the scaled peak. The bar plots show the GO analysis (biological process) of genes associated with H3K9me3 in each chromatin state (right). (**D**) Percentage of H3K9me3 lost peaks (*P*< 0.05) associated genes (closest gene to peak) that do (‘yes’) or do not (‘no’) overlap with up-regulated genes (log_2_foldchange > 1 & *P_adj_* < 0.05). (**E**) Heatmap of H3K9me3 signal at repetitive regions in *Atrx* WT versus KO MPCs derived by SQuIRE analysis of three biologic replicates. Bold indicates elements of interest (see main text). (**F**) IGV tracks show a representative H3K9me3 differential peak at an IAPEzint ERV element. The yellow bar indicates a differential H3K9me3 peak. The green bar shows the mouse IAPEz-int element. Within each sample genotype, each track represents an independent biologic replicate. (**G**) Percentages of each family of TE up-regulated in *Atrx* KO vs WT MPCs based on an rRNA-depletion sequencing dataset. The up-regulated transposable elements were mapped at an individual locus level. (**H**) Percentage of up-regulated genes in polyA-RNA-seq data that annotated by up-regulated TEs (TE method by locus).

To further characterize the regions in which H3K9me3 was lost, we first used ChromHMM to annotate chromatin states across the whole genome using CUT&RUN-derived data for H3K4me3, H3K27ac, H3K9me3 and H3K27me3 in *Atrx* WT MPCs (Figure 3A, Supplementary Figures S4F, S5) (64). We found that H3K9me3 was enriched in a state marked by H3K9me3 alone (State 4, Supplementary Table S9), annotated as heterochromatin, as well as in a small subset of regions marked by H3K27me3 and H3K9me3 (State 3, Supplementary Table S8), annotated as repressed chromatin (Figure 3A). We then compared H3K9me3 CUT&RUN signal within the regions that make up the states containing H3K9me3 in *Atrx* KO and WT MPCs and found that H3K9me3 was reduced in the *Atrx* KO MPCs at these sites (Figure 3B, C). Genes associated with lost H3K9me3 are notably related to cell development and differentiation.

Next, to better understand the consequences of this observation on gene regulation, we examined the connection between H3K9me3 loss and changes in gene expression. Of the 328 significantly upregulated genes in *Atrx* KO MPCs, 19.5% (64/328) were associated with peaks that had significantly less H3K9me3 signal in the ATRX deficient line (Figure 3D). GO pathway analysis of these genes revealed enrichment of gene sets related to adipocyte differentiation, including lipid localization, fat cell differentiation, and brown fat cell differentiation (Supplementary Table S10). These genes included key adipogenic regulators and markers such as *Fabp4* and *Pparγ*, which are part of the fat cell differentiation GO gene set (Supplementary Table S10). In addition, another gene in the same gene set, *Lpl*, has roles in adipogenic metabolism by mediating hydrolysis of circulating lipoprotein particles (65). Examination of integrated genomic viewer (IGV) tracks in a putative *Fabp4* regulatory element demonstrates H3K9me3 depletion in *Atrx* KO cells (Supplementary Figure S4G).

These observations indicate that ATRX deficiency reduced the heterochromatic histone mark H3K9me3 at genomic regions that were associated with genes relevant to linage-specific differentiation and development. These data suggest that H3K9me3 loss may contribute to gene expression changes in ATRX deficient MPCs, leading to their enhanced capacity for adipogenic differentiation.

### Atrx KO reduces H3K9me3 at ERV repetitive regions

ATRX and the ATRX-associated H3K9 methyltransferase, SETDB1, promote H3K9me3 deposition and silencing at repetitive elements (5). These sequences include transposable elements (TEs) such as endogenous retroviral elements (ERVs), which are a type of long terminal repeat derived from integrated retroviral elements (66). De-repression of ERVs can lead to formation of dsRNA, which is detected by sensors that in turn stimulate innate immune signaling, which has been implicated as a mediator of antitumor immune response (67–70). Separately, ERVs can act as gene regulatory elements with enhancer-like features and as transcription factors binding sites to regulate gene expression (71–73). Given that H3K9me3 is depleted in heterochromatin regions in *Atrx* KO MPCs, we sought to determine how this affected ERV expression and gene regulation in this system.

We compared H3K9me3 enrichment at repetitive elements between *Atrx* KO and WT MPCs. We found that H3K9me3 signal was reduced by deletion of ATRX on multiple types of TEs, including ERV family members (ERV1, ERVK), *Alu*, *ID* and *B2* (Figure 3E). To further define the relationship between H3K9me3 loss at repetitive elements and differentiation phenotypes in *Atrx* KO lines, we mapped the repetitive elements onto the previously defined chromatin states (Figure 3A). While most of repetitive elements were in quiescent (State 6) regions devoid of detectable chromatin marks (Supplementary Figure S6A), outside of these regions ERV1 and ERVK elements preferentially mapped to heterochromatin regions where H3K9me3 was reduced in *Atrx* KO versus WT MPCs (State 4) (Supplementary Figure S6B).

To identify specific subfamilies of ERVs where H3K9me3 is depleted in *Atrx* KO MPCs, we analyzed the H3K9me3 differential peaks by adapting SQuIRE analysis, a RNA-seq analysis pipeline that provides a quantitative and locus-specific information on TE expression (34). The top reduced regions of H3K9me3 in both *Atrx* KO lines is an IAP element (Supplementary Figure S7A, B, Supplementary Tables S11, S12), which is a member of the ERVK family. Loss of H3K9me3 in *Atrx* KO versus WT at a representative region containing an ERVK (IAPEzint) element can be appreciated by review of IGV tracks (Figure 3F).

To determine if TEs are transcriptionally de-repressed in the setting of ATRX-dependent H3K9me3 reduction, we performed RNA-sequencing following rRNA-depletion and analyzed locus-specific repetitive element differential expression between *Atrx* KO and WT MPCs using SQuIRE. We identified 116 significant (*P*_adj_ < 0.05) upregulated TEs in common between both *Atrx* KO clones (Supplementary Table S13). Among these upregulated TEs, 42 (36.2%) belong to the ERV superfamily (Figure 3G), which is the largest subset of upregulated TEs.

To investigate whether de-repressed TEs correlated with nearby gene expression, we analyzed the intersection of upregulated TEs with significantly upregulated genes. We found that the 116 significant upregulated TEs were annotated by 136 genes (using the TE transcript by locus method, Supplementary Table S14). Among those genes, 29 genes (21.3%) were significantly upregulated as measured by RNA-seq (Figure 3H). The adipocytic lineage transcription factor, *Pparg*, was observed in this gene set, raising the possibility that the associated TE could serve as a regulatory element for *Pparg*. In addition, we observed 445 significantly downregulated TEs near 163 genes, of which 30 genes were also downregulated (Supplementary Figure S7C, Supplementary Table S15). These results demonstrate that *Atrx* KO leads to reduction of H3K9me3 on specific TEs including those in regions which potentially regulate an adipogenesis master regulator.

### Loss of ATRX promotes chromatin accessibility and enrichment of active histone modifications at developmental genes

ATRX regulates euchromatin as well as heterochromatin (74), leading us to investigate whether the dysregulation of developmental transcriptional programs in MPCs that we see in *Atrx* KO was connected to changes in active histone modifications, such as H3K4me3 and H3K27ac. We hypothesized that loss of ATRX leads to gain of H3K4me3 in specific promoters and H3K27ac in enhancer-like regions. First, we investigated the distribution of H3K4me3 and H3K27ac in *Atrx* WT and KO MPCs and identified genomic features of differential peaks of H3K4me3 and H3K27ac in *Atrx* KO. As expected, H3K4me3 gained peaks in *Atrx* KO clones are highly represented at promoters (Supplementary Figure S8A, Supplementary Table S16). Enrichment of H3K27ac was mainly in introns, distal intergenic regions and promoters in *Atrx* KO lines (Supplementary Figure S8B, C, Supplementary Table S17).

**Figure 4.**
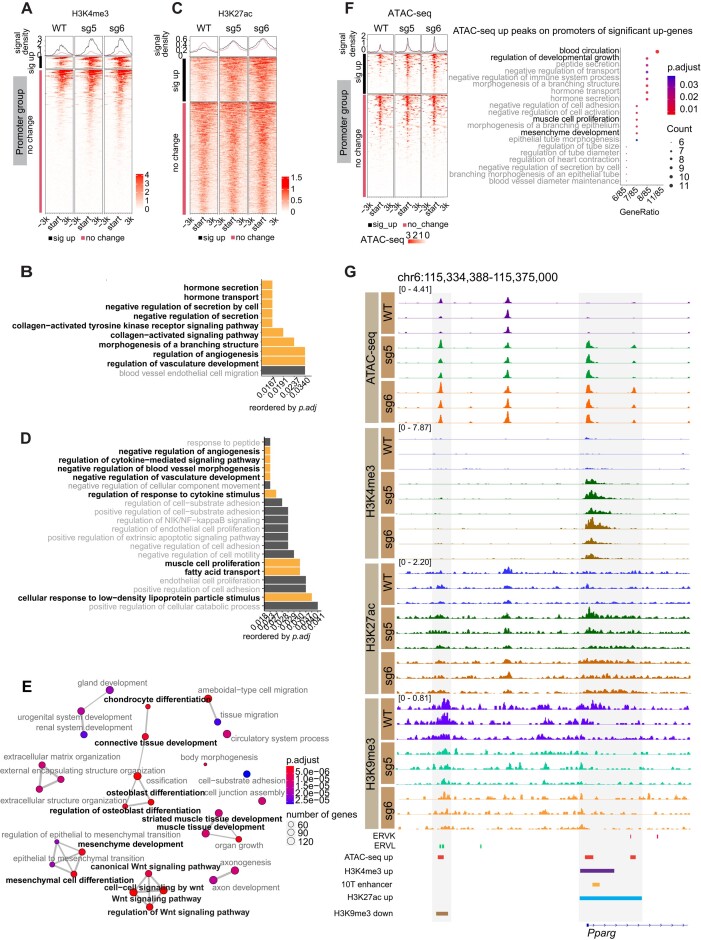
Loss of ATRX alters active chromatin marks and perturbs chromatin accessibility to induce expression of a key adipogenic regulator. (**A**) H3K4me3 signal around promoters of significantly upregulated genes grouped by whether H3K4me3 is gained (black bar) or unchanged (red bar) (**B**) GO terms (biological process) associated with H3K4me3 gained regions. (**C**) H3K27ac signal ±3 kb around the center of H3K27ac peaks near upregulated genes where H3K27ac is gained (black bar) or unchanged (red bar) (**D**) GO analysis (biological process) of H3K27ac significantly increased regions. (**E**) Network plot of gene programs associated with increased accessibility in *Atrx* KO lines. The terms labeled with bold black font indicate those related to the mesenchymal lineage. (**F**) Signal of ATAC-seq ±3 kb around the transcription start site (TSS) of genes that have significantly increased expression in *Atrx* KO MPCs and have an ATAC-seq peak in the TSS region. The regions are grouped based on whether the overlapping ATAC peak has significantly increased signal (black bar) or is unchanged (red bar). The dot plot shows the GO terms associated with upregulated genes. (**G**) IGV tracks of CUT&RUN and ATAC-seq replicates showing a region near the promoter of the *Pparg* gene. Enhancer annotation is from EnhancerAtlas 2.0 database (http://www.enhanceratlas.org/) (82) and converted to the mouse mm10 genome.

Next, we examined the relationship between distribution of H3K4me3 at promoter regions and transcriptional profiles of those genes. Interestingly, less than half of genes upregulated in *Atrx* KO cells gain H3K4me3 at their promoters (Figure 4A) suggesting that other factors may mediate the increased expression in the group where H3K4me3 does not change. Focusing specifically on genes that gain H3K4me3 and are upregulated in an ATRX-dependent fashion, GO pathway analysis revealed genes related to angiogenesis and hormone secretion, the latter of which includes the adipogenesis master regulator, *Pparg* (Figure 4B, Supplementary Table S18).

We also examined the distribution of H3K27ac, focusing on H3K27-gained regions near upregulated genes (Figure 4C). Genes in these regions were enriched in pathways that associated with cell development and fatty acid transport (Figure 4D, Supplementary Table S19). Our data suggests that *Atrx* KO in MPCs leads to changes in both the heterochromatic histone mark H3K9me3 and the euchromatic marks H3K4me3 and H43K27ac. Given the connection of these posttranslational modifications with chromatin compaction, we speculated that ATRX deficiency would lead to a shift in chromatin accessibility. To test this, we performed ATAC-seq, which demonstrated increased accessibility in *Atrx* KO cells at genes associated with mesenchymal development and differentiation (Figure 4E, Supplementary Table S20) (75–77). To investigate the connection of increased chromatin accessibility driven by ATRX deficiency and gene expression changes in *Atrx* KO clones, we separated ATAC-seq peaks at promoters of significantly upregulated genes (Supplementary Table S21) from those at non-promoter regions near or in the same genes (Supplementary Table S22) (Figure 4F). Similar to H3K4me3, while a minority of the upregulated genes showed increased accessibility at their promoters (Figure 4F), these genes were associated with development. The observation that a group of genes increased expression but did not gain chromatin accessibility suggests, as is in the analogous case of upregulated genes that did not gain H3K4me3, that indirect mechanisms may account for transcriptional changes upon deletion of ATRX. In addition, we performed motif analysis for regions with increased chromatin accessibility in *Atrx* KO MPCs. The top motifs include binding sites for a zinc finger protein (ZNF384), AP-1 family members (Jun, FOS), and Sox family transcription factors, which have a role in mesenchymal development (78–80) (Supplementary Figure S9A, Supplementary Table S23).

To understand the relationship between heterochromatin changes and increased chromatin accessibility in ATRX deficient MPCs, we intersected the ATAC-seq gained peaks (8750 peaks) that overlapped with regions showing decreased H3K9me3 (5074 peaks) and identified 162 overlapping regions (Supplementary Figure S9B). Despite this small number of overlapping peaks, GO analysis suggested that this subset was highly representative of programs related to development including in along the mesenchymal lineage (Supplementary Figure S9C, Supplementary Table S24). These results demonstrate that the increased chromatin accessibility coupled with a reduction in H3K9me3 at specific genes in *Atrx* KO MPCs is associated with altered gene expression and a pro-differentiation phenotype in *Atrx* KO MPCs.

### ATRX deficiency induces an active chromatin state at the promoter and putative regulatory element of the adipogenic transcription factor *Pparg*

ATRX loss leads to an aberrant increase in expression of *Pparg* (Figure 1E), which is an important adipogenic transcription factor (81). In addition, in functional assays for adipocytic differentiation, PPARγ protein levels are markedly induced in *Atrx* KO MPCs immediately after induction of adipogenic differentiation (Figure 2B). In order to understand how these phenotypes are functionally linked to the epigenetic changes driven by ATRX deficiency, we investigated ATRX-dependent changes in the chromatin state near the *Pparg* gene. In *Atrx* KO MPCs the promoter of *Pparγ* showed increased accessibility and increased H3K4me3 and H3K27ac, suggesting a more active chromatin state (Figure 4G). Notably, previous work has identified a *Pparg* enhancer element in this same region (82) (Figure 4G, Supplementary Table S25), suggesting multiple mechanisms by which these chromatin changes could enhance *Pparg* expression. In addition, we observed a loss of H3K9me3 and increased accessibility at an ERVL element 20 kb upstream from the *Pparg* promoter, raising the possibility that it functions as an ATRX-dependent gene regulatory element for *Pparg*.

### ATRX associates with accessible and active chromatin in MPCs

To understand whether direct ATRX binding could influence the gene expression and chromatin changes observed in *Atrx* KO MPCs, we mapped ATRX binding sites using CUT&RUN. At telomeric regions, where ATRX is known to function with DAXX to deposit H3.3 and bind to G-quadruplex structures (6), ATRX signal was decreased in both knockout clones compared to WT (*P* < 0.05) (Supplementary Figure S10A). As an additional control, we detected ATRX peaks at zinc finger gene clusters (Supplementary Figure S10B), where ATRX is known to bind (83) that were lost in the *ATRX* KO MPC.

**Figure 5.**
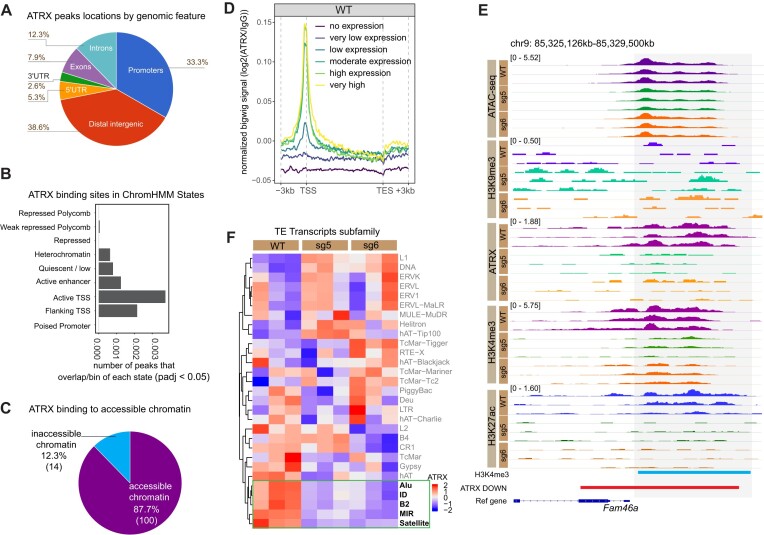
ATRX binding to active chromatin. (**A**) ATRX binding sites by genomic feature. (**B**) Overlap of ATRX binding sites with chromatin states excluding state 5 (artifact/blacklist). x-axis indicates the level of ATRX binding calculated by: (number of ATRX peaks that overlap state bins)/(total bins with that state). (**C**) The percentage of ATRX peaks that overlap ATAC-seq peaks. (**D**) ATRX signal near TSS in gene groups binned based on expression levels. The genes were grouped as follows: ‘no expression’ indicates that TPM (transcripts per million) = 0, ‘very low expression’ indicates the quintile of TPM between 0 and 20%, ‘low expression’ indicates the quintile of TPM between 20 and 40%, ‘moderate expression’ indicates the quintile of TPM between 40 and 60%, ‘high expression’ indicates the quintile of TPM between 60 and 80%, and ‘very high expression’ indicates the quintile of TPM between 80 and 100%. (**E**) IGV tracks at the *Fam46a* gene region. (**F**) Heatmap of ATRX signal over repetitive elements (n=3).

ATRX enrichment at the sites of active transcription start sites (TSS) was recently reported in lymphoblastoid cell lines, suggesting that ATRX has an important role in euchromatin where it regulates gene expression in addition to its more established role at heterochromatin (74). This is consistent with MPC ATRX peaks identified here (*n* = 114; *P*_adj_ < 0.05) (Supplementary Figure S10C, Supplementary Tables S26, S27), where we observed that of the 114 ATRX binding sites, 33.3% were located at promoter regions, and 38.6% were in distal intergenic regions (Figure 5A). The remainder associated with 5′UTRs (5.3%), exons (7.9%), 3′UTRs (2.6%) and introns (12.3%). Mapping ATRX binding sites onto the ChromHMM-defined chromatin states revealed an association with active transcription start sites (TSS, State 8) (Figure 5B). Comparing the ATRX binding sites to *Atrx* WT ATAC-seq peaks, we found that 87.7% (100/114) of ATRX binding sites overlapped with chromatin accessible regions (Figure 5C, Supplementary Table S28). ATRX binding corresponded to H3K27ac enriched regions in 56.1% (64/114) of cases (Supplementary Figure S10D, Supplementary Table S29). Interestingly, in the intersection of H3K27ac occupied regions with ATRX binding sites, 70.3% (45/64) were in non-promoter regions, suggesting a potential role for a subset of ATRX at active enhancers in MPCs (Supplementary Figure S10E, Supplementary Table S30).

Given that ATRX peaks were often localized to open chromatin and active promoters, we explored the relationship between ATRX binding and gene expression. Genes were binned based on expression and ATRX signal was quantified around the TSS of genes in each group (Figure 5D). The most highly expressed genes had the greatest ATRX signal, whereas non-expressed genes had no ATRX enrichment at the TSS. For example, *Fam46a*, which is known to downregulated in differentiating adipocytes (84) and was significantly downregulated in *Atrx* KO MPCs (Supplementary Figure S10F) has an ATRX binding site at its promoter region (Figure 5E). This association of ATRX with active genes is similar to a recent report in a hematopoietic lineage (74) and suggests that ATRX may play a role in maintaining and potentially establishing gene expression in the mesenchymal cells. However, given that only a subset of ATRX binding sites correlate with changes in gene expression in *Atrx* KO MPCs (Supplementary Table S27), there are likely other mechanisms by which ATRX regulates transcription. One possibility may be an indirect mechanism via binding and chromatin-mediated regulation of transposable elements which in turn influence transcription. In support of this possibility, we observed that ATRX binds several families of repetitive elements, including SINEs (*Alu*, *ID*, B2, MIR) and Satellite DNAs (Figure 5F), a finding that was confirmed by a second analysis method (Supplementary Figure S10G). In addition, the ATRX binding sites were enriched for binding motifs of specific transcriptional regulators, such as REST (85), PBX3 (86), and NFYC/A (87,88) (Supplementary Figure S10H).

### ATRX loss in undifferentiated pleomorphic sarcoma leads to epigenetic changes and de-repression of TEs

**Figure 6.**
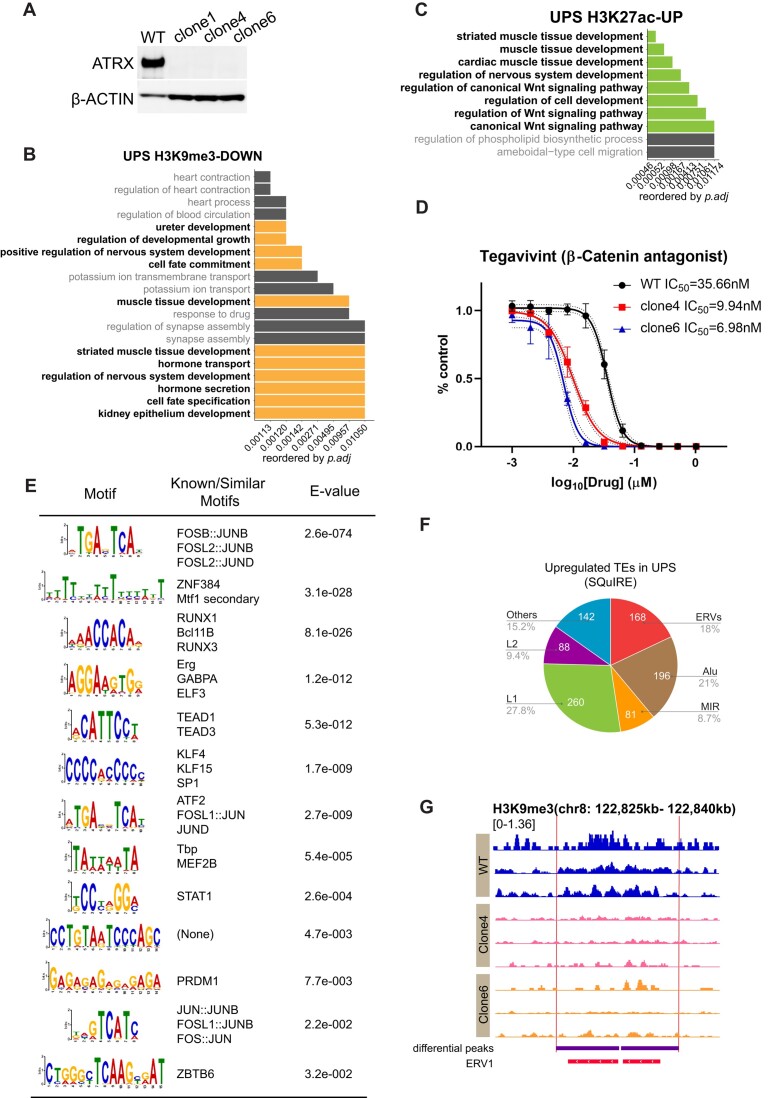
Undifferentiated pleomorphic sarcoma cells lines deficient for ATRX have dysregulated chromatin, de-repress TEs, and are sensitive to Wnt pathway inhibition. (**A**) Immunoblot demonstrating ATRX protein loss in UPS lines. β-ACTIN acts as loading control. (**B**) The GO analysis (biological process) for significant (*P* ≤ 0.05) H3K9me3-lost regions. (**C**) The GO analysis (biological process) for significant (*P* ≤ 0.01) H3K27ac-up regions. (**D**) Dose–response curve for UPS viability normalized to vehicle control following 72 h of treatment with tegavivint. Three biological replicates were performed. The log(IC_50_)s are significantly different for each cell line (*P* < 0.0001, *F*test). Dashed lines, 95% confidence interval; error bars, standard deviation. (**E**) Motif analysis for UPS ATAC-up peaks in *ATRX* KO clones using MEME. (**F**) Percentages of each family of TEs upregulated in *ATRX* KO versus WT UPS. The upregulated transposable elements were mapped at an individual locus level. (**G**) IGV tracks show a representative H3K9me3 differential peak at an ERV1 element. The purple bars indicate differential H3K9me3 peaks. The red bars show a human ERV1 element. Within each sample genotype, each track represents an independent biologic replicate.

To investigate the epigenetic consequences of ATRX deficiency in a human mesenchymal malignancy, we used a patient-derived undifferentiated pleomorphic sarcoma (UPS) cell line (29) to establish *ATRX* KO clones and a WT isogenic control (Figure 6A, Supplementary Figure S11A). UPS are deficient for ATRX in up to third of cases (8–10), making UPS a clinically relevant context to study ATRX loss in cancer. The parental UPS line is p53 deficient with retained RB1 at the protein level, which reflects one of several UPS genotypes (8,29).

To understand the epigenetic role for ATRX in sarcomas, we performed an analysis of H3.3, H3K9me3 and H3K27ac landscapes in two UPS *ATRX* KO clones and a WT control and determined chromatin accessibility by ATAC-seq. As in the MPC model, ATRX loss resulted in an expected significant depletion of H3.3 signal at telomeres (Supplementary Figure S11B). H3K9me3 peaks were significantly lost and gained in the *ATRX* KO clones and were enriched near genes in pathways relevant for development (Figure 6B, Supplementary Figure S11C, Supplementary Tables S31, S32), which is similar to our observation in ATRX deficient mesenchymal progenitor cells (Supplementary Figure S4D, E). Notably, we observed more pathways specific for mesenchymal development in the UPS lines, perhaps reflecting the degree of lineage commitment in the UPS cell of origin, which is yet to be defined.

The active chromatin mark, H3K27ac, was significantly gained in the *ATRX* KO UPS in regions associated with development, particularly in mesenchymal pathways (Figure 6C, Supplementary Table S33), which also similar to MPCs (Figure 4D). This includes the Wnt pathway, which is an important regulator of mesenchymal differentiation (89) and which gained accessible chromatin upon ATRX loss in the MPC context (Figure 4E). To explore the functional consequence of this epigenetic change, we treated ATRX deficient versus retained UPS with tegavitint, a β-Catenin antagonist, to disrupt Wnt signaling. The *ATRX* KO UPS clones had 3.5- to 5-fold lower IC_50_s (9.9 and 6.9 nM) compared to the WT control (35 nM) suggesting a differential dependency on Wnt signaling based on ATRX status (Figure 6D). Notably, tegavivint is being investigated in a therapeutic trial in mesenchymal malignancies (NCT04851119).

In UPS, loss of ATRX led to increased chromatin accessibility, including at genes related to development (Supplementary Figure S11D, Supplementary Table S34). A motif analysis of these newly accessible chromatin regions demonstrated putative binding sites for AP-1 and zinc finger family transcription factors among the most significant predictions (Figure 6E). This is concordant with the most significant predicted binding sites newly accessible regions in MPCs with ATRX loss (Supplementary Figure S9A), suggesting a similar role for ATRX in both contexts. The gained accessibility at AP-1 sites in both the UPS and MPC contexts was particularly notable since deficiency of SETDB1, an H3K9 methyltransferase that interacts with ATRX, leads to opening of chromatin in multiple cell lines at AP-1 motifs at TEs (90).

Transcriptionally, ATRX deficiency in UPS resulted in the significant upregulation of 539 genes and downregulation of 566 downregulated genes in common between the two clones compared to wildtype (Supplementary Figure S12A, B). Gene set enrichment analysis highlighted pathways involved in cell cycle regulation and mitosis among upregulated genes and in genes involved in immune response among downregulated genes (Supplementary Figure S12C, Supplementary Table S35). Notably, similar pathways were downregulated in an ATRX deficient (versus *Atrx* WT) mouse model of UPS (91) suggesting that while ATRX has an important role in regulating transcription in both MPC and UPS, the specific consequences of ATRX deficiency may depend on context, potentially including concurrent p53 loss of function, an event shared in both the human cell lines (29) and mouse UPS (91).

In MPCs, ATRX loss was associated with upregulation of TE expression leading us to additionally focus on TE expression in the mesenchymal malignancy context. In *ATRX* KO UPS compared WT, there were 935 significantly upregulated TE transcripts present in common between clones, which include 168 ERVs (Figure 6F, G, Supplementary Table S36). These results were similar observations in MPCs (Figure 3E–G). To additionally validate the association of ATRX loss and TE de-repression in the UPS context, we analyzed RNA-seq data from a study reporting a mouse model of ATRX deficient versus retained UPS (91). TEs were upregulated in the *Atrx* KO tumors, with the ERV superfamily, including ERV1, ERVL and ERVK, predominating (Supplementary Figure S12D, Supplementary Table S37). Together, these data support an important role for ATRX in maintaining the repression of TEs in both the mesenchymal progenitor and mesenchymal malignancy contexts.

## Discussion

The mesenchymal lineage gives rise to connective tissues. Sarcomas, which are cancers of connective tissues, have recurrent loss of ATRX in up to 30% of specific subtypes, suggesting the potential importance of ATRX deficiency in sarcoma biology and possibilities for precision medicine approaches to treatment (8). However, while ATRX loss is known to contribute to the alternative lengthening of telomere phenotype in sarcomas, the functions of ATRX in the epigenetic regulation of gene expression and downstream processes in the mesenchymal context is less understood.

**Figure 7.**
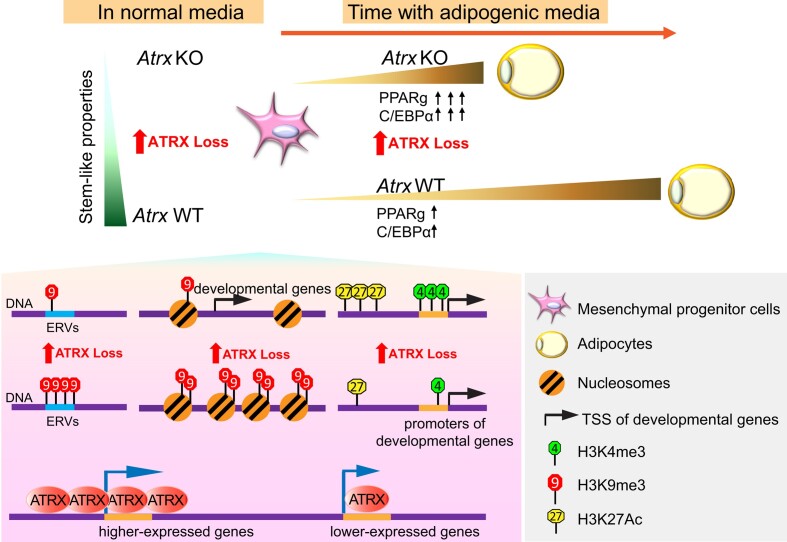
Model for ATRX-dependent chromatin and gene regulation in MPCs. *Atrx* KO MPCs demonstrate reduced stem-like properties, such as slower proliferation, reduced colony formation and downregulated mesenchymal stemness gene expression. In adipogenic media, *Atrx* KO MPCs exhibit accelerated adipocytic differentiation and expression of adipogenic transcription factors. We propose a potential model in which these phenotypes are regulated by multiple epigenetic mechanisms at developmental genes including loss of H3K9me3 near promoters and at ERVs with putative *cis-*regulatory functions, gain of chromatin accessibility, and enrichment of active histone marks. In addition, loss of direct binding of ATRX, which is associated with high levels of genes expression, may contribute. Together, these mechanisms could explain gene expression changes in ATRX deficient MPCs, which in turn mediate differentiation in the mesenchymal lineage context.

Given the important role for ATRX regulating epigenetic states and the intersection of epigenetics with development, we hypothesized that loss of ATRX would affect cell commitment in mesenchymal progenitor cells. We demonstrated that the *Atrx* KO MPCs were sensitive to differentiation induction and exhibited reduced progenitor properties. At the transcriptional level, ATRX loss increased expression of adipogenesis regulators and markers, including *Pparg*, which is an early and direct master regulator of adipogenesis (81). We then investigated ATRX mediation of these phenotypes through epigenetic changes, with a focus on several potential and non-exclusive mechanisms (Figure 7).

First, ATRX deficiency promotes chromatin accessibility and increased transcription at genes associated with mesenchymal differentiation including *Pparγ* and *Fabp4*. Whether ATRX regulates chromatin accessibility directly or indirectly remains an interesting question to be addressed in future work. Second, the loss of ATRX leads to changes in histone posttranslational modifications at specific loci, including the heterochromatin mark H3K9me3 and active chromatin marks, H3K4me3 and H3K27ac. The genes associated with H3K9me3-depleted heterochromatin regions in ATRX-deficient cells are relevant for differentiation and development. This suggests that loss of this repressive mark may create a chromatin environment permissive to signals that activate these programs, which is consistent with the accelerated and increased differentiation phenotype observed in *Atrx* KO MPCs following simulation with adipogenic media.

In addition, ATRX deficiency leads to the loss of H3K9me3 at ERVs, which could represent another potential mechanism for the transcriptional changes induced by ATRX loss given that ERVs can serve as *cis-*regulatory elements and have also been shown to change expression during development (92,93). Finally, histone marks associated with active transcription were also altered upon ATRX loss. We observed increased H3K4me3 at promoters and H3K27ac enrichment near significantly upregulated genes. This suggests that increased H3K4me3 and H3K27ac may also contribute to the induction of mesenchymal development programs

Third, ATRX may regulate gene expression through direct interactions with active chromatin. Recent reports in lymphoblastoid cell lines shows that 38% of ATRX binding sites are localized to promoters (74), which is consistent with our results demonstrating that approximately one-third of ATRX binding sites in MPCs occur at promoters. In contrast, ATRX binding sites in murine embryonic stem cells are mainly restricted to distal intergenic regions and gene bodies, with only 1% on promoters (94). While in murine neuroepithelial progenitors, 17% of ATRX binding sites were located in promoters (94). Therefore, ATRX binding sites differ depending on the cell and developmental contexts with a trend towards increased association with promoters in lineage specific progenitors compared to less differentiated stem cells. Our findings demonstrate that ATRX is not only required for maintaining heterochromatin, but that it may also regulate gene expression through binding to active chromatin regions, consistent with this recently discovered role for ATRX. In our system, ATRX binding was associated with increased gene expression. However, whether ATRX occupancy directly drives transcriptional upregulation is unclear since only a subset of genes near ATRX binding sites are downregulated following ATRX loss. We speculate that ATRX binding might regulate gene expression changes through a distal enhancer mechanism in the case of ATRX binding sites that do not influence expression of nearby genes. In support of this, ATRX signal was decreased at several types of SINEs in *Atrx* KO MPCs, including *Alu*, *B2*, *B4*, *ID* and MIR, which act as transcription factor binding sites (*Alu*(95), MIR (96)) or harbor promoter-like (*B2*) (97) or enhancer-like features (*B4)* (98), respectively.

Our findings demonstrate that ATRX restricts differentiation in mesenchymal progenitor cells. Deletion of ATRX leads to aberrant de-repression of the lineage-restricted transcriptome, accompanied by changes in chromatin accessibility and histone post-translation modifications near transposable elements and at specific genes associated with differentiation. We observed similar changes in histone modifications and chromatin accessibility in a patient-derived UPS cell in which we knocked out ATRX. At the transcriptional level, ATRX deficient UPS cells upregulated TEs, including ERVs, a finding we also observed in a re-analysis of datasets from a mouse model of ATRX deficient UPS (91). In addition, epigenetic changes in ATRX deficient UPS led us to uncover a selective sensitivity of ATRX deficient UPS cell to Wnt pathway inhibition by tegavivint. This warrants further preclinical investigation in UPS, particularly since tegavivint is being evaluated in clinical trials for patients with mesenchymal tumors. While we would not expect full concordance between the sequelae of ATRX loss in the mesenchymal progenitor and the transformed mesenchymal malignancy context, the overall effects on epigenetic dysregulation and de-repression of TEs was similarly observed in both settings.

Our findings raise several questions including whether the changes observed in active histone marks, chromatin accessibility, and de-repression of ERVs after *Atrx* KO occur through direct or indirect mechanisms and how these epigenetic changes contribute to the pathogenesis or may invoke therapeutic opportunities in sarcomas in which ATRX loss is common (e.g., UPS and leiomyosarcoma). One intriguing possibility is that ATRX loss creates a permissive chromatin and transcriptional context in which mesenchymal progenitors are susceptible to subsequent sarcomagenic events such as loss of *TP53* and *RB1* (8) or the aberrant expression of specific transcription factors. Overall, results presented herein expand our understanding of the epigenetic consequences of ATRX function in in the mesenchymal context.

## Supplementary Material

gkae160_Supplemental_Files

## Data Availability

All genomic and transcriptional data of MPCs and human UPS cells were deposited in the Gene Expression Omnibus (GEO) repository under accession number GSE240030. Code is available in Zenodo at https://zenodo.org/doi/10.5281/zenodo.10668665.
