# Supplementary material for: ATRX guards against aberrant differentiation in mesenchymal progenitor cells
Source: Nucleic Acids Res. 2024 Mar 13;52(9):4950–68. doi: 10.1093/nar/gkae160 (PMC11109985; doi:10.1093/nar/gkae160)
Supplement: gkae160_Supplemental_Files [file gkae160_supplemental_files.zip › Fang_Supplementary Figure-with legends_NAR-02835-X-2023.pdf]

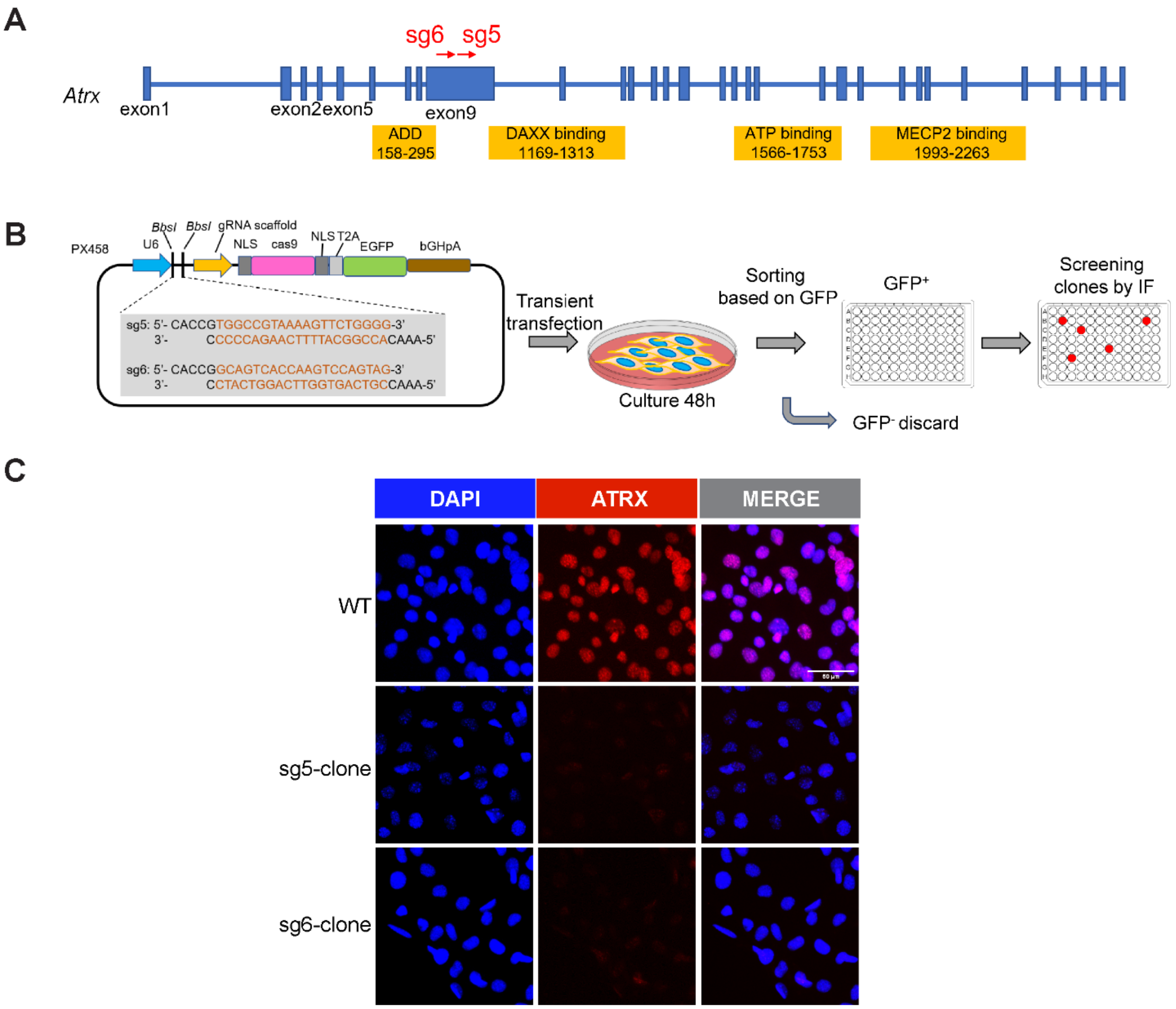

Supplementary Figure S1: Generating ATRX knockout in C3H/10T1/2 cells. (A) Schematic of the of mouse *Atrx* gene structure. The yellow boxes indicate *Atrx* domains; blue boxes are the exons. The red arrows show the sgRNA target locations. **(B)** Workflow for establishing ATRX KO MPCs. The plasmid containing cas9 and sgRNAs targeting *Atrx* (WT control used an empty vector) was transiently transfected in cells. After 48 h, single cells expressing GFP were seeded in a 96 well plate (one cell per well). After 10 days, the cells were screened for ATRX expression (see panel **C**). Knockout clones from two different sgRNA were used in downstream experiments. **(C)** Immunofluorescence staining of ATRX in *Atrx* KO vs WT isogenic pairs. The scale bar is 50 μm.

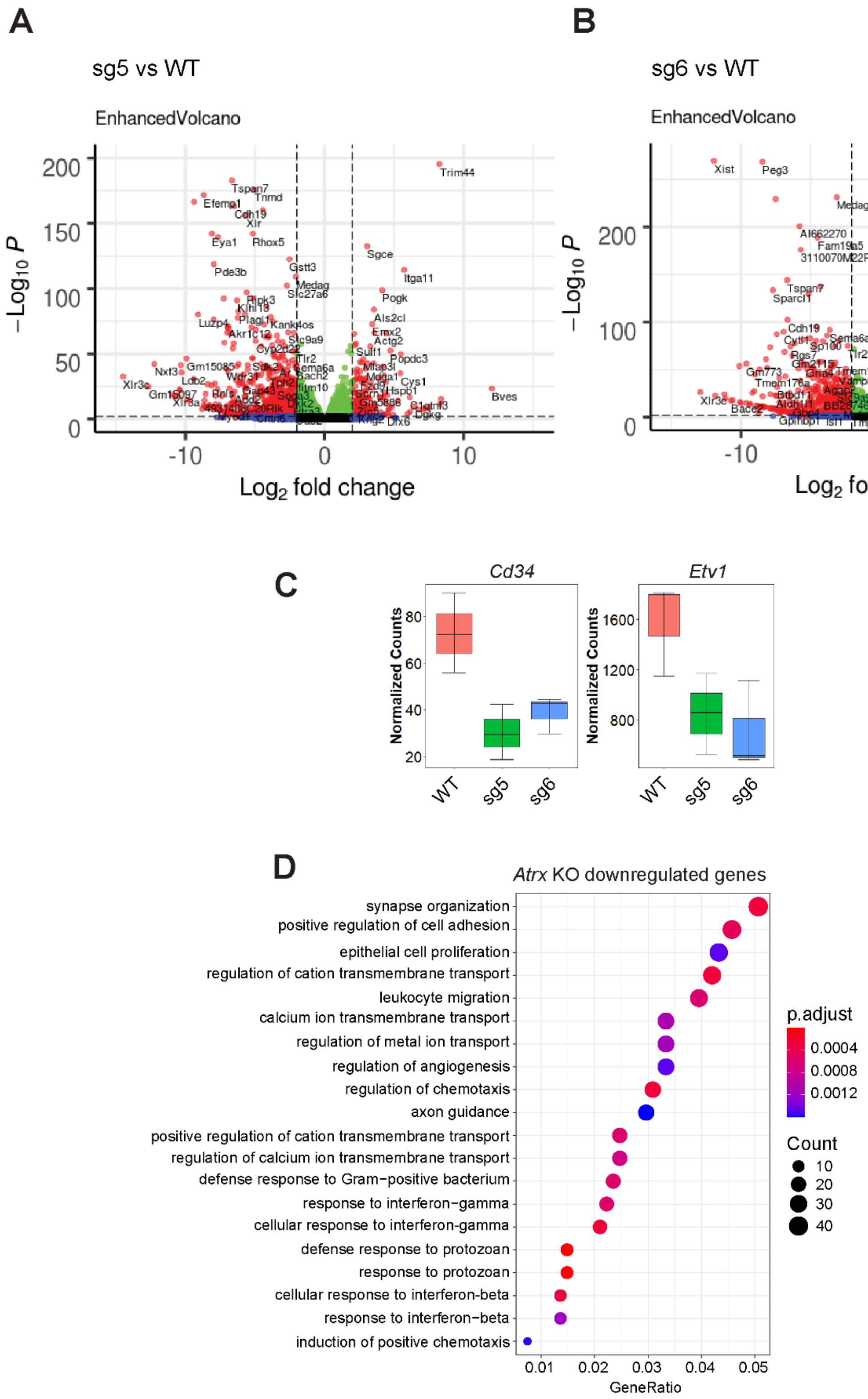
A
sg5 vs WT
EnhancedVolcano
$-Log_{10}$ P
Log_2 fold change
B
sg6 vs WT
EnhancedVolcano
$-Log_{10}$ P
Log_2 fold change
C
Cd34
Etv1
Normalized Counts
WT
sg5
sg6
D
Atrx KO downregulated genes
synapse organization
positive regulation of cell adhesion
epithelial cell proliferation
regulation of cation transmembrane transport
leukocyte migration
calcium ion transmembrane transport
regulation of metal ion transport
regulation of angiogenesis
regulation of chemotaxis
axon guidance
positive regulation of cation transmembrane transport
regulation of calcium ion transmembrane transport
defense response to Gram−positive bacterium
response to interferon−gamma
cellular response to interferon−gamma
defense response to protozoan
response to protozoan
cellular response to interferon−beta
response to interferon−beta
induction of positive chemotaxis
GeneRatio
p.adjust
Count

Supplementary Figure S2: Loss of ATRX significantly perturbs the transcriptome of mesenchymal progenitor cells. (A) and **(B)** Volcano plots of each *Atrx* KO clone. The red points show genes which log_2foldchange > 2 or < -2 and p <0.01. The blue points indicate genes with log_2foldchange > 2 or < -2 and p value > 0.01. Green points indicate genes with log_2foldchange between -2 to 2 and p <0.01. Black dots indicate genes with log_2foldchange between -2 to 2 and p >0.01. **(C)** Boxplots for transcripts of interest. **(D)** GO analysis (biological process) for significantly down-regulated genes. Cutoffs used were log_2foldchange < -1, and p_{adj} < 0.05.

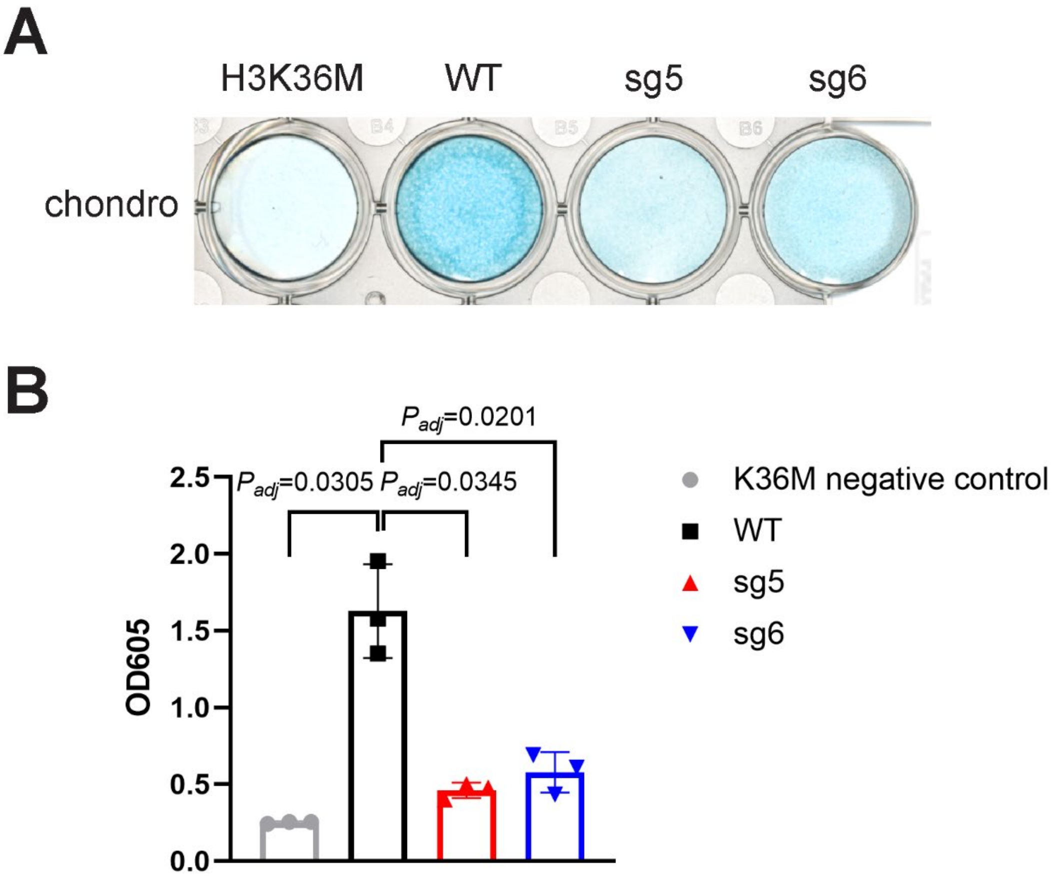

Supplementary Figure S3: ATRX deficiency inhibits MPC differentiation into chondrocytes. **(A)** Alcian blue staining for chondrocytes. chondro: chondrocyte differentiation media treatment. **(B)** Quantification of chondrocyte differentiation. The y-axis shows the optical density (OD) of the solubilized Alcian blue after chondrocyte staining. K36M indicates the H3K36M oncohistone negative control. Data from three biological replicates are plotted with each point representing individual value from each replicate *p.adj* was calculated by a post hoc comparison to WT following a paired one-way ANOVA .

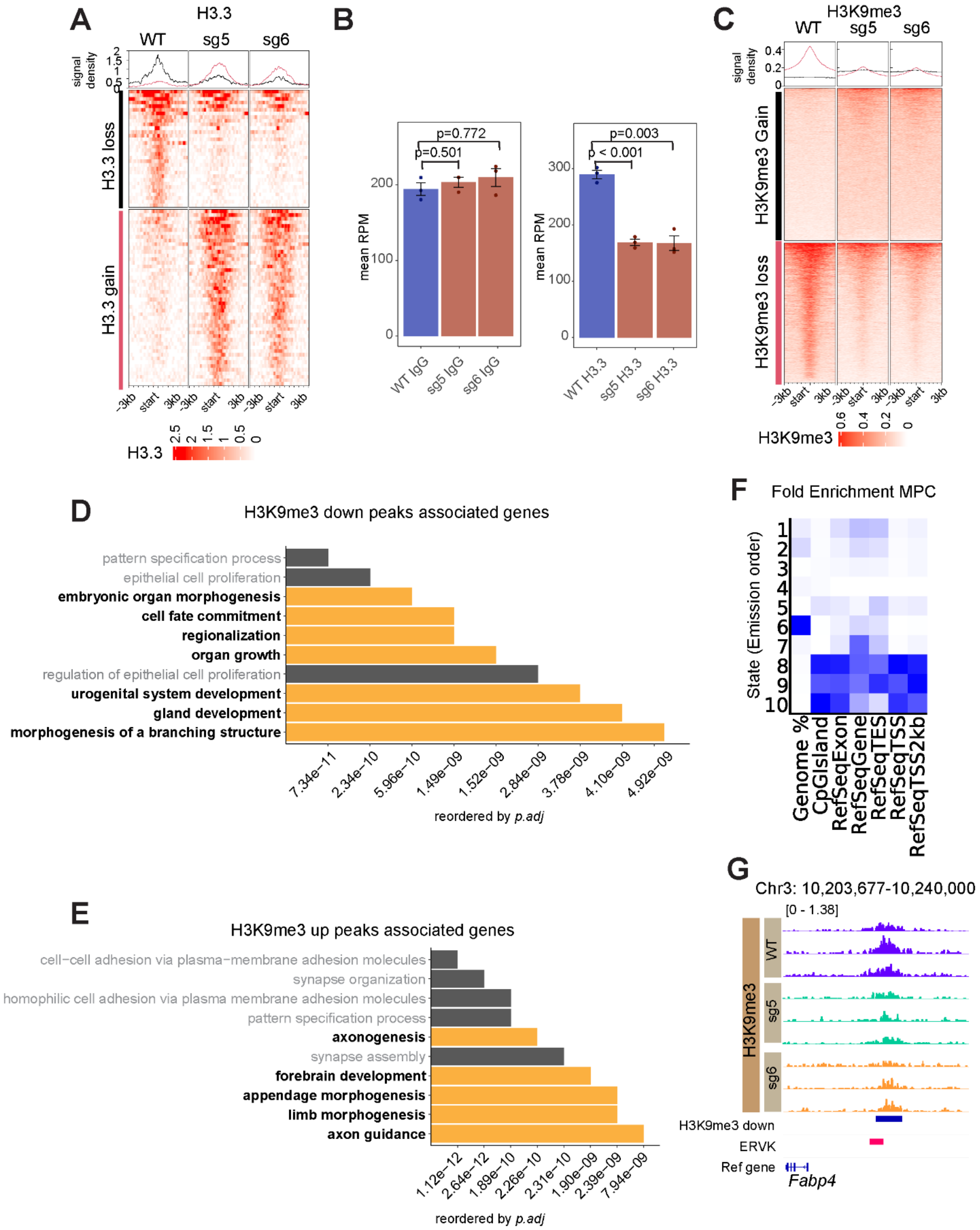
A
H3.3
WT
sg5
sg6
signal density
H3.3 loss
H3.3 gain
-3kb
start
3kb
H3.3
2.5
2
1.5
1
0.5
0
B
p=0.772
p=0.501
mean RPM
200
100
0
WT IgG
sg5 IgG
sg6 IgG
p=0.003
p < 0.001
300
WT H3.3
sg5 H3.3
sg6 H3.3
C
H3K9me3
H3K9me3 Gain
H3K9me3 loss
0.4
0.2
0.6
H3K9me3
D
H3K9me3 down peaks associated genes
pattern specification process
epithelial cell proliferation
embryonic organ morphogenesis
cell fate commitment
regionalization
organ growth
regulation of epithelial cell proliferation
urogenital system development
gland development
morphogenesis of a branching structure
7.34e−11
2.34e−10
5.96e−10
1.49e−09
1.52e−09
2.84e−09
3.78e−09
4.10e−09
4.92e−09
reordered by p.adj
E
H3K9me3 up peaks associated genes
cell−cell adhesion via plasma−membrane adhesion molecules
synapse organization
homophilic cell adhesion via plasma membrane adhesion molecules
pattern specification process
axonogenesis
synapse assembly
forebrain development
appendage morphogenesis
limb morphogenesis
axon guidance
1.12e−12
2.64e−12
1.89e−10
2.26e−10
2.31e−10
1.90e−09
2.39e−09
7.94e−09
reordered by p.adj
F
Fold Enrichment MPC
State (Emission order)
1
2
3
4
5
6
7
8
9
10
Genome %
CpGIsland
RefSeqExon
RefSeqGene
RefSeqTES
RefSeqTSS
RefSeqTSS2kb
G
Chr3: 10,203,677-10,240,000
[0 - 1.38]
H3K9me3
WT
sg5
sg6
H3K9me3 down
ERVK
Ref gene
Fabp4

Supplementary Figure S4: *Atrx* KO MPCs gain and loose H3.3 and H3K9me3 at specific loci. (A) Tornado plot of H3.3 signal at differential regions. **(B)** IgG and H3.3 signal at telomeric regions. RPM reads assigned per million mapped reads. The error bars show the standard deviations. The points show the data from individual biological replicates. *p* values were calculated using an unpaired one-way ANOVA with Tukey's method for post hoc comparison. **(C)** H3K9me3 on differential regions. **(D)** The GO analysis (biological process) for significant ($p \leq 0.05$) H3K9me3-lost regions or **(E)** H3K9me3-gained regions associated genes. **(F)** Average genome coverage and annotation of genic and non-genic elements in chromatin states determined by ChromHMM. **(G)** The Integrative genomic viewer tracks show H3K9me3 signals on *Fabp4* gene region. The blue bar shows the H3K9me3 significant down peaks (p-value < 0.05. The pink bar shows mouse ERVK elements.

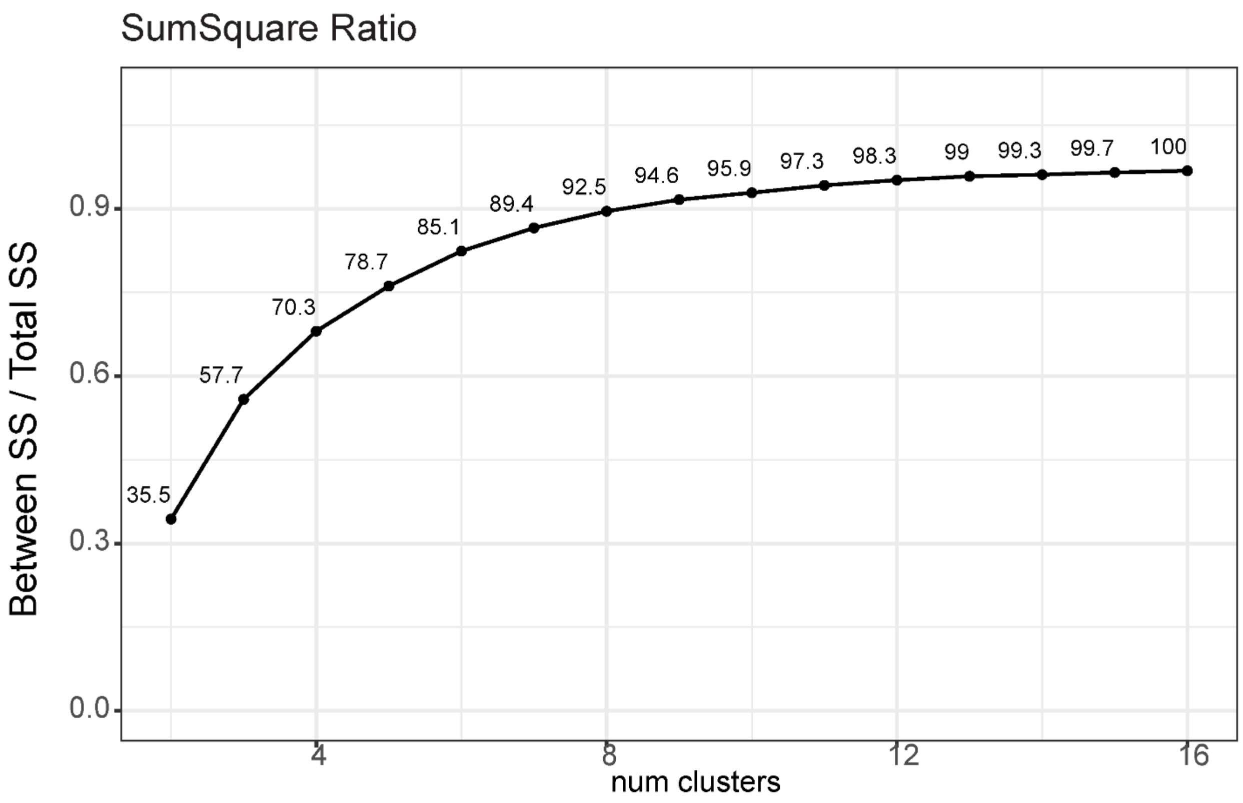

Supplementary Figure S5: ChromHMM model. We established chromatin states using a multivariate hidden Markov model (ChromHMM) (64). The plotted line demonstrates k-means clustering of the emission probabilities from *Atrx* WT model. We used the number of states that k-means equals to or higher than 95%. SS, sum of squares.

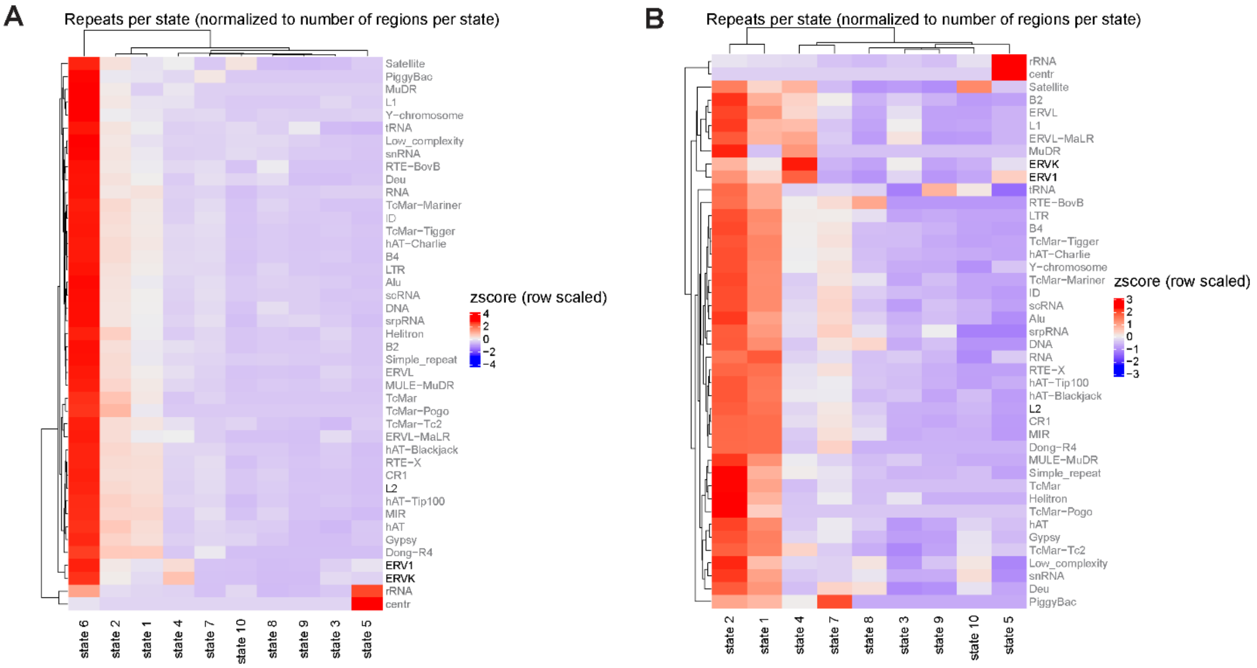

Supplementary Figure S6: Enrichment of repetitive elements in chromatin states. (A) Heatmap of enrichment of repetitive elements in all ChromHMM-derived10 states. **(B)** Heatmap of enrichment of repetitive elements in 9 states excluding quiescent regions (state 6).

A

H3K9me3 SQuIRE
sg5 vs WT
(Log$_2$foldchange < -0.5 & p_{adj} < 0.05)

Repeat elements	log2FoldChange	padj
IAPA_MM-int:ERVK:LTR	-0.816496869	0.039844
IAPEz-int:ERVK:LTR	-0.793516217	0.001854
IAPLTR1_Mm:ERVK:LTR	-0.721368856	0.042289
ERVB2_1-I_MM-int:ERVK:LTR	-0.662394548	0.019362

B

H3K9me3 SQuIRE
sg6 vs WT
(Log$_2$foldchane < -0.5 & p_{adj} < 0.05)

Repeat elements	log2FoldChange	padj
IAPLTR1_Mm:ERVK:LTR	-0.821470048	0.005677
IAPEz-int:ERVK:LTR	-0.802215015	0.00232
IAPA_MM-int:ERVK:LTR	-0.788499776	0.033545
IAPLTR2a2_Mm:ERVK:LTR	-0.700587849	0.020823
IAPLTR1a_Mm:ERVK:LTR	-0.696345914	0.02403
IAPLTR2_Mm:ERVK:LTR	-0.652599169	0.040852
RLTR4_MM-int:ERV1:LTR	-0.585097342	0.024024

C

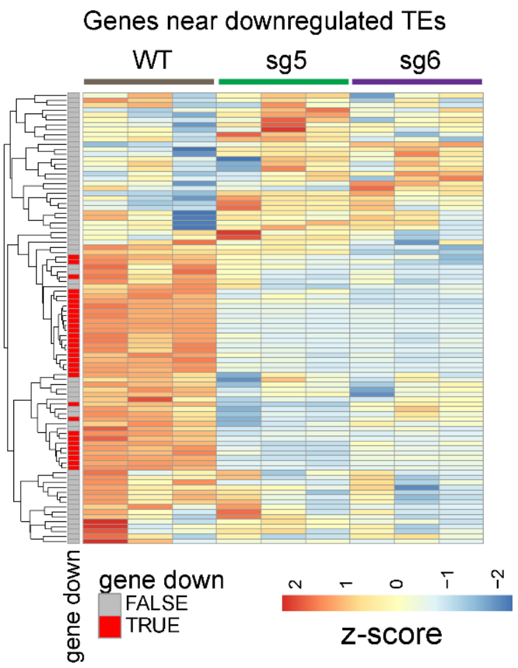

Supplementary Figure S7: The association of H3K9me3 and TE expression. TEs associated with H3K9me3 loss (log$_2$foldchange < -0.5 with p_{adj} < 0.05) in each of two *Atrx* KO clones **(A)** and **(B)** vs WT MPCs. **(C)** The heatmap shows gene expression near downregulated TEs (TRUE: log$_2$foldchange < -1 with p_{adj} < 0.05).

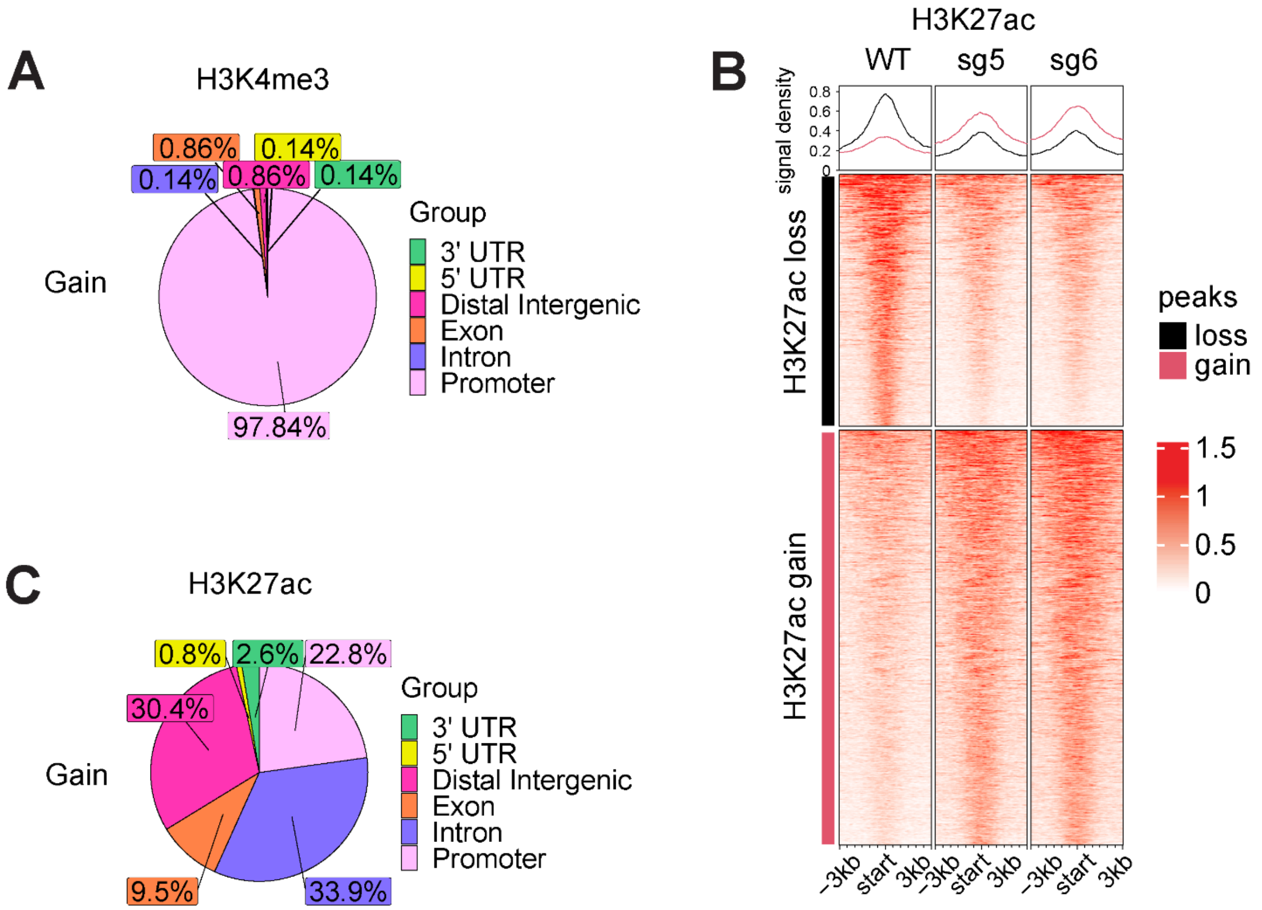

Supplementary Figure S8: ATRX deficiency alters the distribution of active chromatin marks and chromatin accessibility. (A) Relative distribution of H3K4me3 gained peaks in *Atrx* KO MPCs by genomic feature. **(B)** Tornado plot of H3K27ac at differential regions. **(C)** Relative distribution of H3K27ac gained peaks in *Atrx* KO MPCs by genomic features.

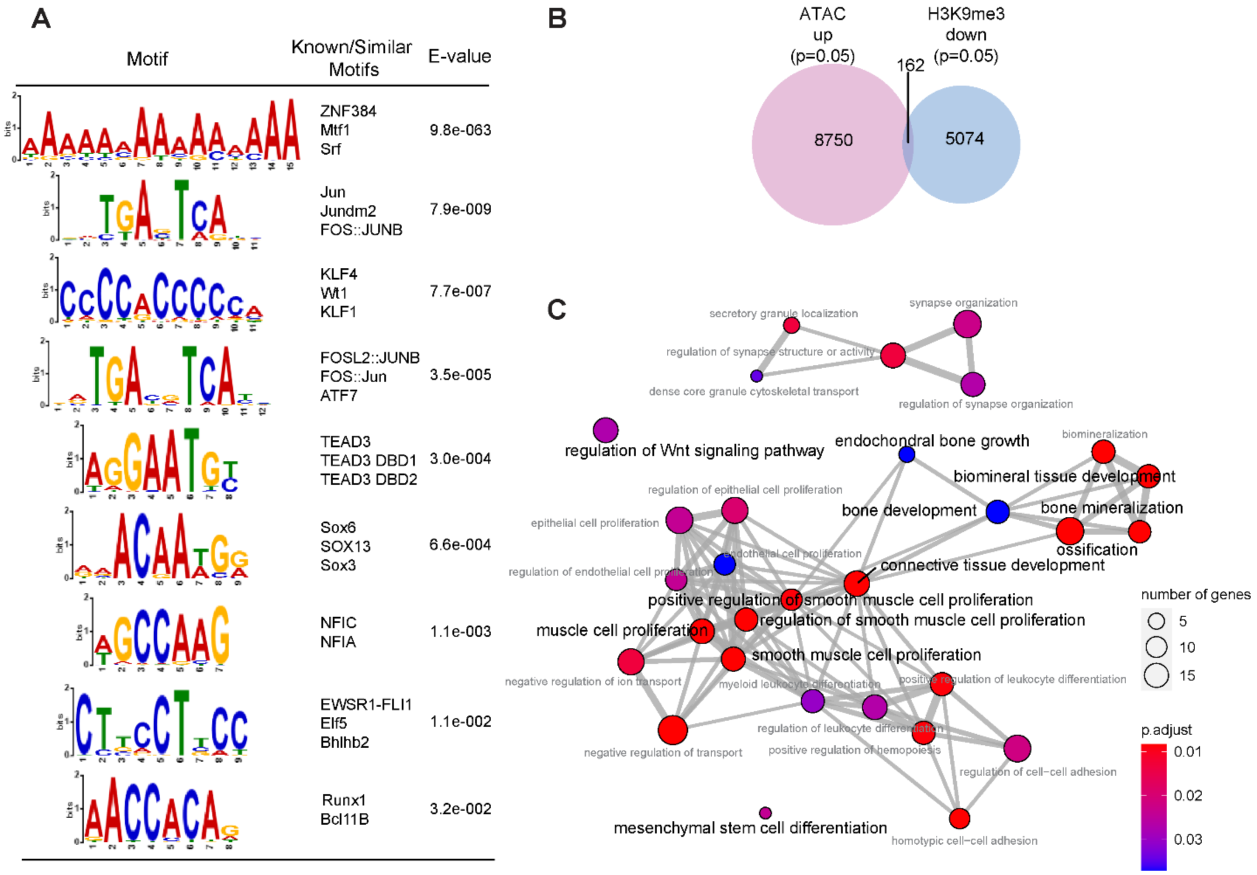

Supplementary Figure S9: H3K9me3 depleted regions that gain chromatin accessibility are enriched in developmental genes in *Atrx* KO MPCs. (A) Motif analysis (MEME) of ATAC peaks gained from ATRX deficiency. **(B)** The intersection of ATAC-seq gained with lost H3K9me3 regions in *Atrx* KO versus WT MPCs. **(C)** Network plot of gene sets associated with the intersecting peaks in (A). Only significant (p_{adj}<0.05) terms are shown.

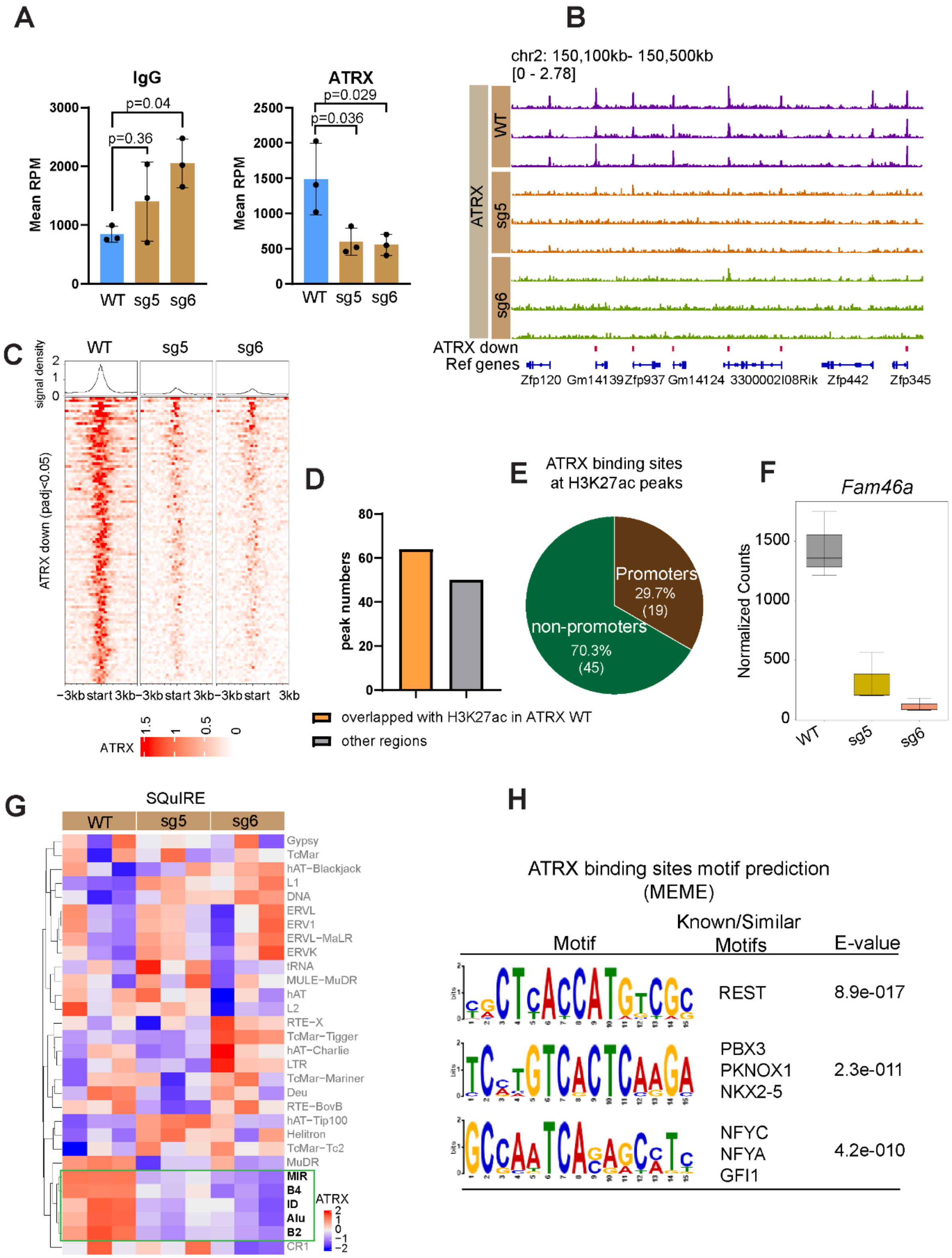

A
IgG
ATRX
Mean RPM
p=0.04
p=0.36
p=0.029
p=0.036
WT sg5 sg6
B
chr2: 150,100kb- 150,500kb
[0 - 2.78]
ATRX
WT
sg5
sg6
ATRX down
Ref genes
Zfp120 Gm14139 Zfp937 Gm14124 3300002I08Rik Zfp442 Zfp345
C
signal density
ATRX down (padj<0.05)
−3kb start 3kb
ATRX
D
peak numbers
overlapped with H3K27ac in ATRX WT
other regions
E
ATRX binding sites at H3K27ac peaks
Promoters 29.7% (19)
non-promoters 70.3% (45)
F
Fam46a
Normalized Counts
G
SQuIRE
Gypsy
TcMar
hAT−Blackjack
L1
DNA
ERVL
ERV1
ERVL−MaLR
ERVK
tRNA
MULE−MuDR
hAT
L2
RTE−X
TcMar−Tigger
hAT−Charlie
LTR
TcMar−Mariner
Deu
RTE−BovB
hAT−Tip100
Helitron
TcMar−Tc2
MuDR
MIR
B4
ID
Alu
B2
CR1
H
ATRX binding sites motif prediction (MEME)
Motif
Known/Similar Motifs
E-value
REST
8.9e-017
PBX3
PKNOX1
NKX2-5
2.3e-011
NFYC
NFYA
GFI1
4.2e-010

Supplementary Figure S10: ATRX binds to repetitive elements in heterochromatin and to active regions in euchromatin (A) ATRX and IgG signal at telomeric regions. The bar plots show IgG and ATRX signals on telomeres, respectively. The error bars show the standard deviations. The points show the data from individual biological replicates. *p* values were calculated using an unpaired one-way ANOVA with Tukey's method for post hoc comparison. RPM (Reads per million mapped reads) assigned per million mapped reads. **(B)** Integrative genomic viewer tracks show typical regions of ATRX peaks (pink bars) at Zinc finger gene clusters. **(C)** ATRX signal at peak regions that have significantly higher signal in *Atrx* WT vs KO cells (p_{adj} ≤0.05). **(D)** The numbers of ATRX binding sites overlapped with H3K27ac enriched peaks in WT group. **(E)** The percentage of ATRX binding sites at H3K27ac-enriched regions in *Atrx* WT cells. **(F)** Boxplot of normalized counts of *Fam46a* transcripts in MPC lines based on RNA-seq. **(G)** SQuIRE analysis shows that ATRX signals are reduced on specific repetitive elements. **(H)** Motif analysis (MEME) of ATRX binding sites in MPCs.

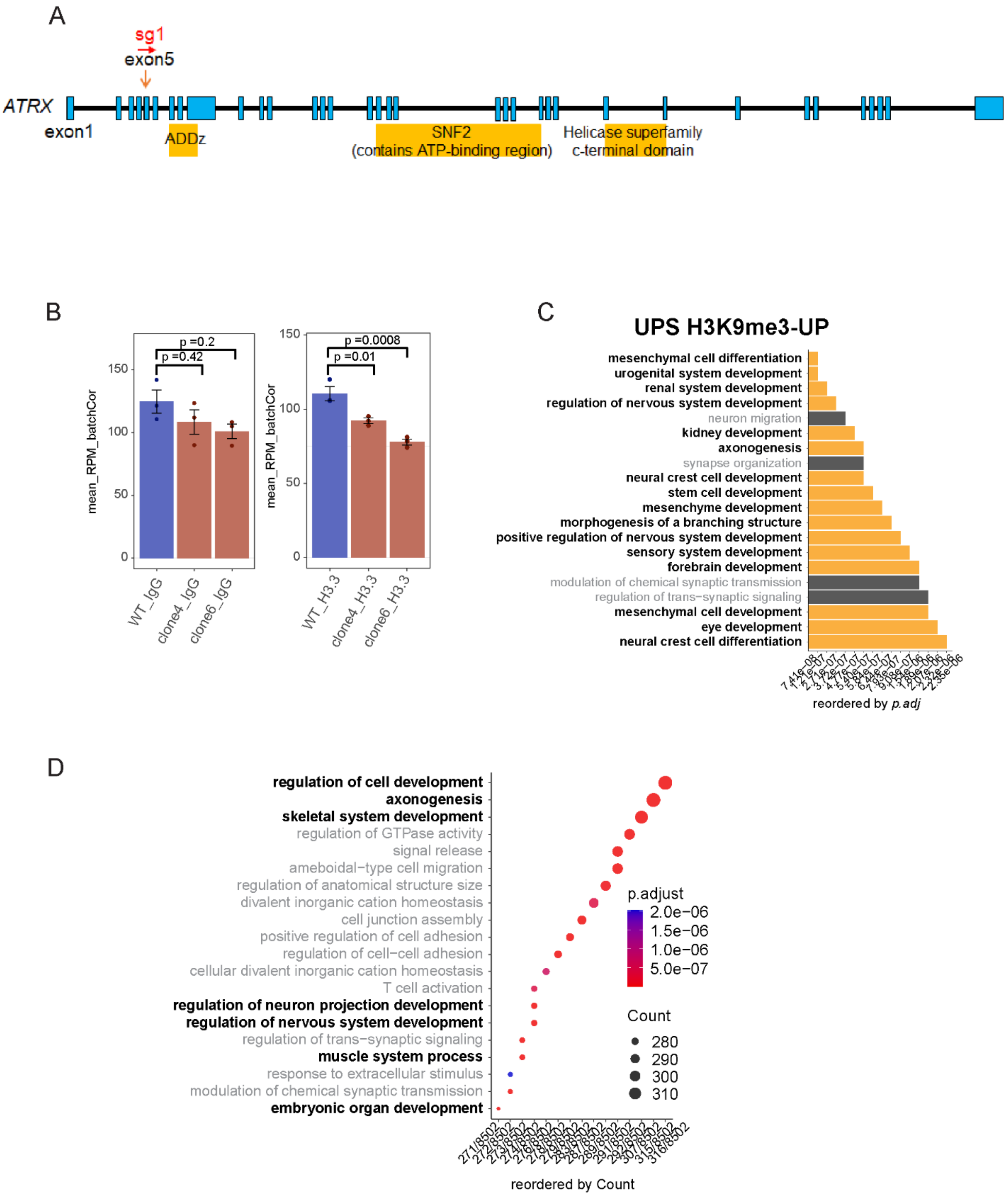
A
sg1
exon5
ATRX
exon1
ADDz
SNF2
(contains ATP-binding region)
Helicase superfamily
c-terminal domain
B
p =0.2
p =0.42
mean_RPM_batchCor
150
100
50
0
WT_IgG
clone4_IgG
clone6_IgG
p =0.0008
p =0.01
WT_H3.3
clone4_H3.3
clone6_H3.3
C
UPS H3K9me3-UP
mesenchymal cell differentiation
urogenital system development
renal system development
regulation of nervous system development
neuron migration
kidney development
axonogenesis
synapse organization
neural crest cell development
stem cell development
mesenchyme development
morphogenesis of a branching structure
positive regulation of nervous system development
sensory system development
forebrain development
modulation of chemical synaptic transmission
regulation of trans−synaptic signaling
mesenchymal cell development
eye development
neural crest cell differentiation
reordered by p.adj
D
regulation of cell development
axonogenesis
skeletal system development
regulation of GTPase activity
signal release
ameboidal−type cell migration
regulation of anatomical structure size
divalent inorganic cation homeostasis
cell junction assembly
positive regulation of cell adhesion
regulation of cell−cell adhesion
cellular divalent inorganic cation homeostasis
T cell activation
regulation of neuron projection development
regulation of nervous system development
regulation of trans−synaptic signaling
muscle system process
response to extracellular stimulus
modulation of chemical synaptic transmission
embryonic organ development
p.adjust
2.0e−06
1.5e−06
1.0e−06
5.0e−07
Count
280
290
300
310
reordered by Count

Supplementary Figure S11. CUT&RUN and ATAC-seq analysis of *ATRX* KO versus WT UPS lines. (A) Schematic of the of human *ATRX* gene structure. The yellow boxes indicate *ATRX* domains; blue boxes are the exons. The red arrows show the sgRNA target location. **(B)** IgG and H3.3 signal at telomeric regions. RPM reads assigned per million mapped reads. The error bars show the standard deviations. The points show the data from individual biological replicates. *p* values were calculated using an unpaired one-way ANOVA with Tukey's method for post hoc comparison. **(C)** The GO analysis (biological process) for significant ($p \leq 0.05$) H3K9me3-gained regions associated genes. **(D)** Bar plot of gene programs associated with increased accessibility in *ATRX* KO UPS lines. The terms labeled with bold black font indicate those related to development.

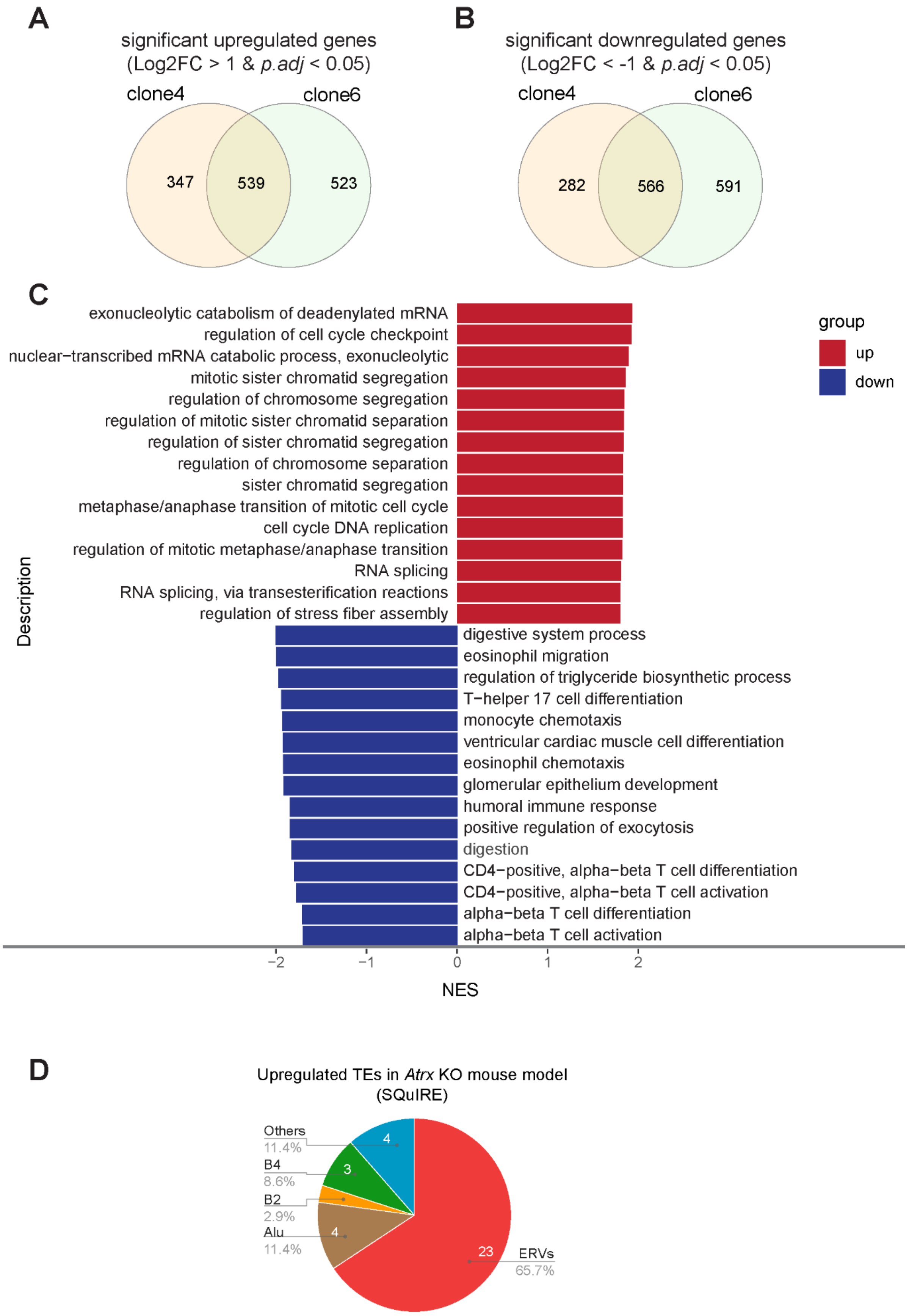

A
significant upregulated genes
(Log2FC > 1 & p.adj < 0.05)
clone4
clone6
347
539
523
B
significant downregulated genes
(Log2FC < -1 & p.adj < 0.05)
clone4
clone6
282
566
591
C
exonucleolytic catabolism of deadenylated mRNA
regulation of cell cycle checkpoint
nuclear−transcribed mRNA catabolic process, exonucleolytic
mitotic sister chromatid segregation
regulation of chromosome segregation
regulation of mitotic sister chromatid separation
regulation of sister chromatid segregation
regulation of chromosome separation
sister chromatid segregation
metaphase/anaphase transition of mitotic cell cycle
cell cycle DNA replication
regulation of mitotic metaphase/anaphase transition
RNA splicing
RNA splicing, via transesterification reactions
regulation of stress fiber assembly
digestive system process
eosinophil migration
regulation of triglyceride biosynthetic process
T−helper 17 cell differentiation
monocyte chemotaxis
ventricular cardiac muscle cell differentiation
eosinophil chemotaxis
glomerular epithelium development
humoral immune response
positive regulation of exocytosis
digestion
CD4−positive, alpha−beta T cell differentiation
CD4−positive, alpha−beta T cell activation
alpha−beta T cell differentiation
alpha−beta T cell activation
Description
−2
−1
0
1
2
NES
group
up
down
D
Upregulated TEs in Atrx KO mouse model
(SQuIRE)
Others
11.4%
4
B4
8.6%
3
B2
2.9%
Alu
11.4%
4
23
ERVs
65.7%

Supplementary Figure S12. Transcriptomic changes in ATRX deficient UPS. (A) The intersection of significantly upregulated genes (log_2foldchange > 1, p_{adj} < 0.05) or **(B)** significantly downregulated genes (log_2foldchange < -1, p_{adj} value < 0.05) in both human UPS *ATRX* KO clones based on polyA-selected RNA-seq datasets. **(C)** Bar plot of GSEA gene ontology (GO) (biological process) analysis for significant upregulated and downregulated genes from (A) and (B). The red bars show upregulated pathways, and the blue bars show downregulated pathways. The NES indicates the normalized enrichment scores. All shown pathways are significant (p_{adj} < 0.05). **(D)** Percentages of each family of TE up-regulated in *ATRX* KO mouse UPS vs WT based on a polyA-selected RNA-seq dataset (88). The upregulated transposable elements were mapped at an individual locus level.
