# Supplementary material for: ATRX guards against aberrant differentiation in mesenchymal progenitor cells
Source: Nucleic Acids Res. 2024 Mar 13;52(9):4950–68. doi: 10.1093/nar/gkae160 (PMC11109985; doi:10.1093/nar/gkae160)
Supplement: gkae160_Supplemental_Files [file gkae160_supplemental_files.zip › Supplementary Table legends.docx]

**Supplementary Table legends**

| Table number | Name of the table |
| --- | --- |
| Supplementary Table S1 | Up genes in both clones with Log_2_foldchange >1. |
| Supplementary Table S2 | Down genes in both clones with Log_2_foldchange < -1 |
| Supplementary Table S3 | GO analysis of up genes (biological process) |
| Supplementary Table S4 | Normalized count numbers of RNA-seq datasets |
| Supplementary Table S5 | GO analysis of down genes (biological process) |
| Supplementary Table S6 | GO analysis of H3K9me3_down peaks (GREAT, *p<*0.05) |
| Supplementary Table S7 | GO analysis of H3K9me3_up peaks (GREAT, *p<*0.05) |
| Supplementary Table S8 | GO analysis of H3K9me3 down peaks in State3 (GREAT) |
| Supplementary Table S9 | GO analysis of H3K9me3 down peaks in State4 (GREAT) |
| Supplementary Table S10 | GO analysis of H3K9me3 down peaks (CS) overlapped with RNA-seq significant up genes |
| Supplementary Table S11 | SQUiRE analysis of H3K9me3 (sg5 vs. WT) |
| Supplementary Table S12 | SQUiRE analysis of H3K9me3 (sg6 vs. WT) |
| Supplementary Table S13 | rRNA-depletion seq_SQUiRE analysis in both clones |
| Supplementary Table S14 | Genes over up TEs in polyA seq and rRNA-dep seq |
| Supplementary Table S15 | Genes over down TEs in polyA seq and rRNA-dep seq |
| Supplementary Table S16 | H3K4me3 genomic features in both clones |
| Supplementary Table S17 | H3K27ac genomic feature in both clones |
| Supplementary Table S18 | GO analysis of H3K4me3 up peaks over upregulated genes (Stringent) |
| Supplementary Table S19 | Go analysis of H3K27ac up peaks over upregulated genes (Stringent) |
| Supplementary Table S20 | GO analysis of ATAC-seq up peaks (*p*<0.001) in both clones |
| Supplementary Table S21 | GO analysis of ATAC up peaks(promoters) over upregulated genes |
| Supplementary Table S22 | GO analysis of ATAC up peaks (non-promoters) over upregulated genes |
| Supplementary Table S23 | MPC ATAC-up motif analysis |
| Supplementary Table S24 | ATAC up peaks over H3K9me3 down peaks (*p<*0.05) |
| Supplementary Table S25 | 10T cell enhancers |
| Supplementary Table S26 | All differential peaks of ATRX without significance filtered |
| Supplementary Table S27 | Significant ATRX binding sites (*p_adj_*<0.05) |
| Supplementary Table S28 | ATRX significant binding sites over ATAC peaks in WT (GREAT) |
| Supplementary Table S29 | ATRX significant binding sites over H3K27ac peaks in WT (GREAT) |
| Supplementary Table S30 | ATRX significant binding sites over H3K27ac non-promoter peaks in WT(GREAT) |
| Supplementary Table S31 | UPS GO analysis of H3K9me3_down peaks (GREAT, *p<*0.05) |
| Supplementary Table S32 | UPS GO analysis of H3K9me3_up peaks (GREAT, *p<*0.05) |
| Supplementary Table S33 | UPS GO analysis of H3K27ac up peaks in both clones (Stringent, *p<*0.01) |
| Supplementary Table S34 | UPS GO analysis of ATAC-seq up peaks in both clones (*p<*0.05) |
| Supplementary Table S35 | UPS DESeq-GSEA-GOBP |
| Supplementary Table S36 | UPS SQuIRE analysis in both clones (*p<*0.05) |
| Supplementary Table S37 | Published mouse UPS RNA-seq dataset SQuIRE analysis |
| Supplementary Table S38 | ATAC-seq index primers |
